# MoNap1, a Nucleosome Assemble Protein 1, Regulates Growth, Development, and Pathogenicity in *Magnaporthe oryzae*

**DOI:** 10.3390/jof9010050

**Published:** 2022-12-28

**Authors:** Shulin Zhang, Yu Wang, Xinyue Cui, Jinmei Hu, Xiaoru Kang, Yuyan Liu, Yuemin Pan

**Affiliations:** 1 Department of Plant Pathology, College of Plant Protection, Anhui Agricultural University, Hefei 230036, China; 2 Anhui Province Key Laboratory of Crop Integrated Pest Management, Anhui Agricultural University, Hefei 230036, China

**Keywords:** *Magnaporthe oryzae*, pathogenicity, appressorium, nutrient transport, cell cycle

## Abstract

Nap1 is an evolutionarily conserved protein from yeast to human and is involved in diverse physiological processes, such as nucleosome assembly, histone shuttling between the nucleus and cytoplasm, transcriptional regulation, and the cell cycle regulation. In this paper, we identified nucleosome assemble protein MoNap1 in *Magnaporthe oryzae* and investigated its function in pathogenicity. Deletion of *MoNAP1* resulted in reduced growth and conidiation, decreased appressorium formation rate, and impaired virulence. MoNap1 affects appressorium turgor and utilization of glycogen and lipid droplets. In addition, MoNap1 is involved in the regulation of cell wall, oxidation, and hyperosmotic stress. The subcellular localization experiments showed that MoNap1 is located in the cytoplasm. MoNap1 interacts with MoNbp2, MoClb3, and MoClb1 in *M. oryzae*. Moreover, deletion of *MoNBP2* and *MoCLB3* has no effects on vegetative growth, conidiation, and pathogenicity. Transcriptome analysis reveals that *MoNAP1* is involved in regulating pathogenicity, the melanin biosynthetic process. Taken together, our results showed that MoNap1 plays a crucial role in growth, conidiation, and pathogenicity of *M. oryzae*.

## 1. Introduction

Nucleosome assembly protein 1 (Nap1) is an integral component in the establishment, maintenance, and dynamics of eukaryotic chromatin [1]. Nap1 family proteins are evolutionarily conserved histone chaperones that play important roles in diverse biological processes [2]. Nap1 was originally identified in HeLa cells as a histone chaperone [3]. Further, homologs of Nap1 have been identified in multiple organisms, such as *Saccharomyces cerevisiae*, *Drosophila melanogaster*, tobacco, and rice [4,5,6]. In *S. cerevisiae*, deletion of *NAP1* leads to alterations in the gene expression of about 10% of the genome [7], but no significant phenotype was observed in *NAP1* knockout yeast strains [8]. On the contrary, a *NAP1* knockout in *D. melanogaster* resulted in either embryonic lethality or poorly viable adults [9]. In plants, different plant NAP1-like proteins display distinct functions in vivo, which was supported by their distinct intracellular localization, e.g., both NtNAP1_L3 and NtNAP1_L4 were localized exclusively in the cytoplasm, whereas OsNAP1_L1 was localized in both the cytoplasm and the nucleus [4].

Previous studies indicated that Nap1 is a chromatin-assembly factor and histone chaperone protein [3,10]. Chromatin assembly involves the initial deposition of a tetramer of histones H3 and H4 onto DNA, followed by the addition of H2A-H2B heterodimers to form the histone octamer [11,12]. In this process, Kap114p is required for nuclear import of H2A and H2B, and Nap1p can bind directly Kap114p [13]. In addition, Nap1 has also been implicated in cell-cycle regulation [14], transcription regulation [15], incorporation and exchange of histone variants [16,17,18], and the promotion of nucleosome sliding [19]. Both yeast and *Xenopus laevis NAP1* as well as *X. laevis SET* have been indicated to interact specifically with B-type cyclins [14,20]. Moreover, there have been reports that the purified Nap1 can be phosphorylated by cyclin B/p34cdc2 kinase complexes that control the transition between the S and G2 phases [8]. In yeast, *NAP1* is indispensable for the function of B-type cyclin Clb2, and the deletion of *NAP1* causes mitotic delays [8,14]. The yeast Nap1(yNap1) also interacts with several other mitotic factors, including the kinase Gin4, Sda1, and Nap1-binding protein 1 (Nbp1) [21,22,23]. These findings suggest that *NAP1* participates in the control of mitotic events. Nap1 can also stimulate the binding of transcription factors, presumably by making DNA more accessible by removing one or both H2A/H2B dimers or by promoting nucleosome sliding [15]. Meanwhile, the C-terminal region of yNap1 is required for the removal of histone H2A/H2B dimers from nucleosomes, and it appears likely that SET uses this mechanism to activate transcription [24]. In *S. cerevisiae*, Nap1-binding protein (Nbp2) was identified to interact with Nap1 by a yeast two-hybrid system [25]. In addition, Nbp2 functioned to promote mitotic cell growth at high temperature and to maintain cell wall integrity. Loss of Nbp2 results in cell death at high temperature and in sensitivity to calcofluor white [25]. Although Nap1 has been widely studied in different species, the function of Nap1 in filamentous fungus has not been researched much yet. 

The blast fungus *M. oryzae* is the most serious disease of cultivated rice and therefore poses a threat to the world’s food security [26]. The importance of rice is underlined by the fact that approximately one-half of the world’s population relies on rice for their main caloric intake [27]. *M. oryzae* infections are initiated when three-celled conidia attach to the hydrophobic rice surface [28]. Next, conidia can germinate within two hours and form germ tube and then develop domed-shaped structure called appressorium [29,30]. The matured appressorium develops a penetration peg and generates the turgor pressure by accumulation of high concentration glycerol to break the rice leaf cuticle and send a narrow hypha into the underlying epidermal cells [31]. Subsequently, the invasive hyphae spread within and between plant cells, resulting in typical lesion and conidiation to spread the disease to adjacent plants [28]. In this study, we used *M. oryzae* as a model to investigate roles of Nap1 in plant pathogenic fungi. We found that MoNap1 not only regulates development, conidiation, and pathogenicity but also affects cell-cycle related pathway. We further identified three MoNap1-interacting proteins, including MoNbp2, MoClb3, and MoClb1. Our findings further provide insights into MoNap1-mediated development and pathogenicity in *M. oryzae*.

## 2. Result

### 2.1. Identification and Subcellular Localization of MoNAP1 in M. oryzae

**Figure 1:**
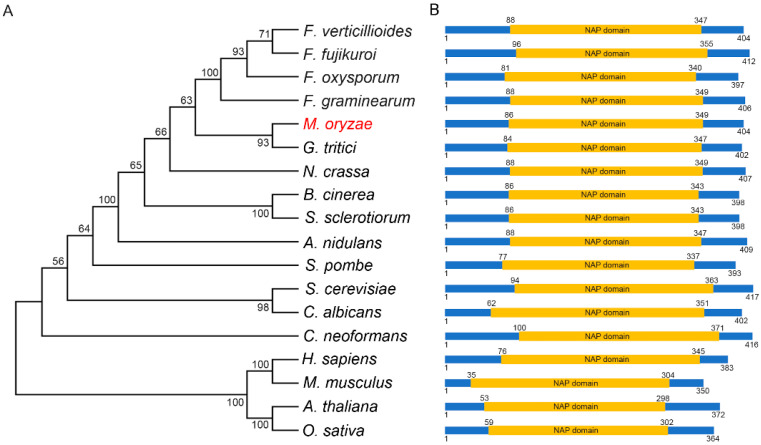
Phylogenetic tree analysis and domain architecture of Nap1 homologues in different organisms. (**A**) Phylogenetic analysis of MoNap1 and its orthologous proteins obtained by Blastp search from fungus, plant, and animal. The protein sequences used for alignment include XP_003709662.1 (*Magnaporthe oryzae*), XP_018753938.1 (*Fusarium verticillioides*), XP_023427587.1 (*Fusarium fujikuroi*), XP_018234142.1 (*Fusarium oxysporum*), XP_011327240.1 (*Fusarium graminearum*), XP_009222249.1 (*Gaeumannomyces tritici*), XP_955998.2 (*Neurospora crassa*), XP_001555380.1 (*Botrytis cinerea*), XP_001598116.1 (*Sclerotinia sclerotiorum*), ABU87403.1 (*Aspergillus nidulans*), NP_587838.1 (*Schizosaccharomyces pombe*), NP_012974.1 (*Saccharomyces cerevisiae*), KAF6071858.1 (*Candida albicans*), XP_571114.1 (*Cryptococcus neoformans*), NP_001294853.1 (*Homo sapiens*), XP_036011785.1 (*Mus musculus*), NP_194341.1 (*Arabidopsis thaliana*), and XP_015639209.1 (*Oryza sativa*). These amino acid sequences were used to construct a phylogenetic tree with the MEGA 6 program. The position of MoNap1 in the phylogenetic tree is indicated by the red font highlighting. (**B**) Domains prediction of MoNap1 and its orthologous proteins was performed using the SMART website (http://smart.embl-heidelberg.de/, accessed on 2 November 2021).

To identify the Nap1 in *M. oryzae*, the amino acid sequence of Nap1 from *S. cerevisiae* was used to run a BLASTp search in EnsemblFungi (http://fungi.ensembl.org/Magnaporthe_oryzae/Info/Index, accessed on 2 November 2021). We identified a single copy of *M. oryzae* specific Nap1 (MoNap1) (XP_003709662.1) encoded by *MGG_06924*. MoNap1 was predicted to encode 404 amino acids, and phylogenetic analysis was carried out using the amino acid sequence of Nap1 from fungus, plant, and animal, indicating Nap1 is conserved in different organisms. MoNap1 is more homologous to Nap1 of *G. tritici* (Figure 1A). Further amino acid alignment results showed that Nap1 protein contains a conserved NAP domain across kingdoms (Figure 1B). Taken together, these results revealed that Nap1 protein is highly conserved across kingdoms. 

**Figure 2:**
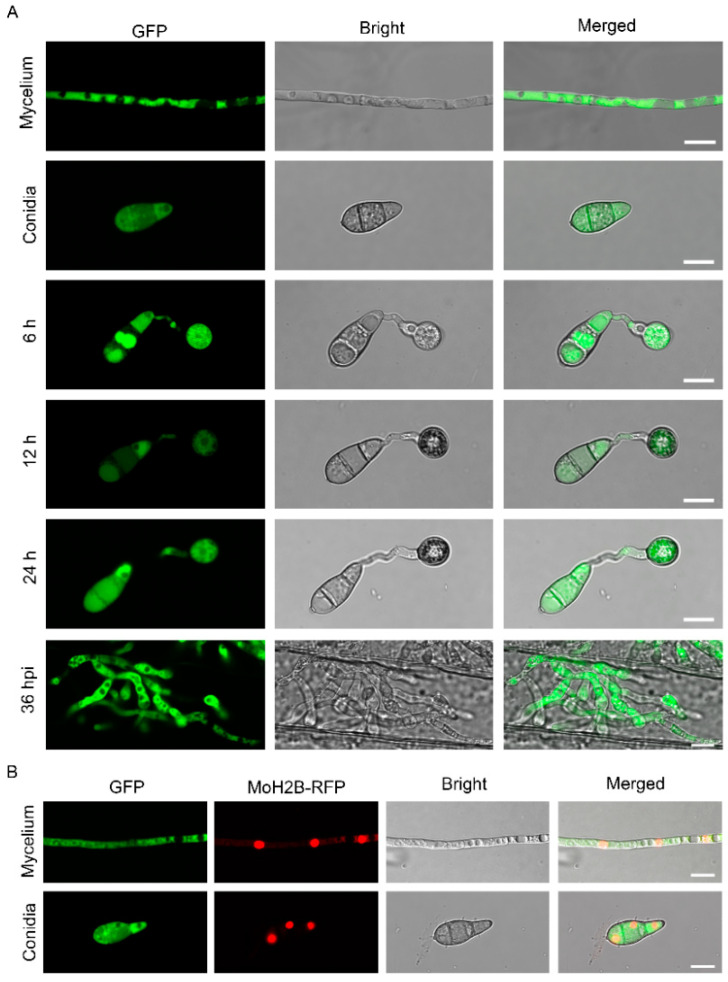
Subcellular localization of MoNap1 at different developmental stages of *M. oryzae*. (**A**) Subcellular localization of MoNap1 in vegetative mycelia, conidia, appressorium, and infected mycelia. Bar = 10 µm. (**B**) MoNap1-GFP and MoH2B-RFP were co-transformed into WT. Conidia and vegetative hyphae co-localization of MoNap1-GFP and MoH2B-RFP was observed under NIKON laser scanning confocal microscope. Bar = 10 µm.

In *S. cerevisiae*, Nap1 has been reported to be a nucleocytoplasmic shuttling protein [8]. To determine the subcellular localization of MoNap1 in *M. oryzae*, the fusion cassette MoNap1-GFP under control of its native promoter was constructed and then co-expressed the MoH_2_B-RFP in the wild-type (WT) strain. We found MoNap1 was mostly located in cytoplasm in conidium, appressorium, vegetative mycelia, and infection mycelia (Figure 2A,B). This result suggested that MoNap1 was localized in cytoplasm during each growth and development stage of *M. oryzae*.

### 2.2. Targeted Gene Deletion and Complementation of MoNAP1

To determine the roles of *MoNAP1* in growth development and pathogenicity of *M. oryzae*, we generated the targeted gene deletion mutant of *MoNAP1* in the wild-type strain Guy11 background using a homologous recombination strategy (Figure S1A). Four putative transformants of the *MoNAP1* deletion were identified by polymerase chain reaction (PCR) using primer pairs (Table S1 and Figure S1B). These transformants were further confirmed by Southern blot assay and reverse transcription polymerase chain reaction (RT-PCR) assay (Figure S1C,D). Corresponding results obtained from these assays showed that *MoNAP1* was successfully replaced with a hygromycin-resistance cassette (*HPH*) to generate *MoNAP1* deletion *ΔMonap1* (#1, #3, #4, and #7). In addition, we also performed a complementation experiment to generate the complementation strains *ΔMonap1/MoNAP1* by introducing the native promoter-driven *MoNAP1* coding region into *ΔMonap1* mutant (#1). The complementation strains *ΔMonap1/MoNAP1* was verified by RT-PCR (Figure S1D) and recovered to WT in basic phenotypic. The *ΔMonap1* mutant (#1) and *ΔMonap1/MoNAP1* were used for assessment in some phenotypes. 

### 2.3. MoNAP1 Is Involved in Vegetative Growth, Melanin Biosynthesis, and Conidia Production

**Figure 3:**
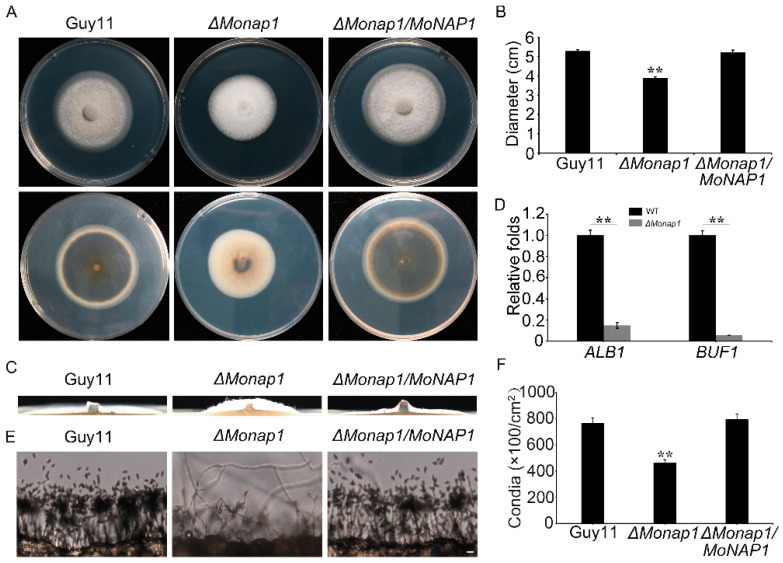
*MoNAP1* is involved in vegetative growth, melanin formation, and asexual reproduction. (**A**) Colony morphology of WT, *ΔMonap1*, and *ΔMonap1/MoNAP1* strains on CM were observed after 7 days at 28 °C. (**B**) The colony diameters were measured and subjected to statistical analysis. (**C**) *ΔMonap1* mutant appears as a thick colony with fluffier aerial mycelia compared to WT and complemented strains. (**D**) Expression level of *MoALB1* and *MoBUIF1* in WT and *ΔMonap1* mutant. The expression level of different genes in WT was set as 1. (**E**) Conidia formation was observed under light microscope after all strains incubating on artificial hydrophobic surface for 24 h at 28 °C. (**F**) Statistical analysis of the sporulation quantity of all strains. For each strain, three independent biological experiments with four replicates each time were carried out. Error bars represent standard deviation, and asterisks above the columns indicate significant differences between WT and *ΔMonap1* estimated by Student’s *t*-test (** *p* < 0.01).

To analyze the roles of *MoNAP1* in vegetative growth, we observed the colony growth and mycelial morphology of the *ΔMonap1* mutant. Compared with WT and the *ΔMonap1/MoNAP1*, we found a significant growth reduction and fluffier aerial mycelia in the *ΔMonap1* mutant (Figure 3A–C). Meanwhile, we noticed that the *ΔMonap1* mutant colony showed less pigment and whiter hyphae compared with WT strain and *ΔMonap1/MoNAP1* after culturing in CM medium for seven days (Figure 3A). This result prompted us to speculate that *MoNAP1* could affect the expression of pigmentation biosynthesis-related genes. As expected, qRT-PCR analysis revealed that *ALB1* and *BUF1* were down-regulated in the mutant *ΔMonap1* in contrast to the WT and *ΔMonap1/MoNap1* strains (Figure 3D).

To determine whether *MoNAP1* plays role in asexual development in *M. oryzae*, the WT strain Guy11, *ΔMonap1*, and *ΔMonap1/MoNAP1* were cultured on RDC medium for 8 days, and then, the number of conidia was counted. We found that conidiation of *ΔMonap1* mutant was drastically reduced compared with the WT strain Guy11 and the complementation strain *ΔMonap1/MoNAP1* (Figure 3F). To validate this result, we further revealed that *MoNAP1* deletion affected the development of conidiophore. We noticed that the number of conidia per conidiophore produced was significantly reduced in the *ΔMonap1* mutant (Figure 3E). These results strongly indicated that the loss of *MoNAP1* causes slower vegetative growth, thicker aerial hyphae, decreased melanin formation, and reduced conidiation. 

### 2.4. MoNAP1 Is Required for M. oryzae Pathogenicity

**Figure 4:**
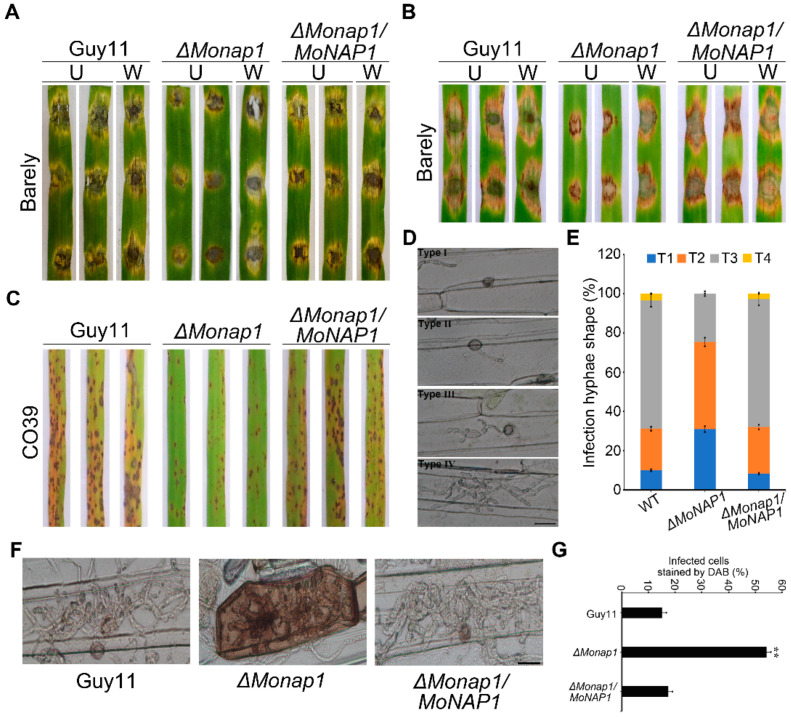
Knockout of *MoNAP1* leads to weaker disease in *M. oryzae*. (**A**) Virulence assay performed on wounded (W) or unwounded (U) barley leaves. Lesions formed on barley leaves inoculated with mycelial blocks of WT, *ΔMonap1*, and *ΔMonap1/MoNAP1* and observed at 5 days post inoculation (dpi). (**B**) Virulence assay performed on wounded (W) or unwounded (U) barley leaves. Lesions formed on barley leaves inoculated with conidial suspensions (1 × 10^5^ conidia/mL) of WT, *ΔMonap1*, and *ΔMonap1/MoNAP1* and observed at 5 dpi. (**C**) Virulence assay performed on rice leaves. Rice seedlings were sprayed with conidial suspensions (1 × 10^5^ conidia/mL) of WT, *ΔMonap1*, and *ΔMonap1/MoNAP1* and observed at 5 dpi. For each strain, three independent biological experiments with four replicates each time were carried out. (**D**) Detached barley leaves were inoculated with conidial suspensions (1 × 10^5^ conidia/mL) from tested strains, and invasive hyphae (IH) formed in barley epidermal cells were observed under light microscope at 36 hpi. Type Ⅰ (T1), only penetration peg without invasive; Type Ⅱ (T2), only one single invasive hypha without branches. Type Ⅲ (T3), with multiple branches but restricted in one cell. Type Ⅳ (T4), extended to neighboring cell. Bar =20 µm. (**E**) Statistical analysis for each infection type. For each strain, three independent biological experiments with four replicates each time were carried out. Error bars represent standard deviation, and asterisks above the columns indicate significant differences between WT and *ΔMonap1* estimated by Student’s *t*-test (** *p* < 0.01). (**F**) Barley epidermis infected by tested strains was stained by 3,3-diaminobenzidine (DAB) and observed under light microscope at 36 hpi. Bar = 20 µm. (**G**) Statistical analysis for proportion of the infected cells stained by DAB. For each strain, at least 100 invaded cells were observed, and the numbers of stained cells were counted. Error bars represent standard deviation, and asterisks above the columns indicate significant differences between WT and *ΔMonap1* estimated by Student’s *t*-test (** *p* < 0.01).

To evaluate the role of *MoNAP1* in the pathogenicity of *M. oryzae*, we conducted pathogenicity assays on different plant hosts. The WT Guy11 and the complementation strain *ΔMonap1/MoNAP1* caused severe lesions, while the *ΔMonap1* mutant produced smaller lesions after inoculated on isolated intact and wounded barley leaves with mycelial agar plugs for 5 days (Figure 4A). After inoculated on detached intact and wounded barley leaves with 1 × 10^5^ conidia ml^−1^ conidial suspensions of Guy11, *ΔMonap1*, and *ΔMonap1/MoNAP1* for 5 days, the *ΔMonap1* mutant also resulted in smaller disease lesions compared to Guy11 and *ΔMonap1/MoNAP1* (Figure 4B). In addition, conidia suspensions (1 × 10^5^ conidia ml^−1^) of tested strains were sprayed onto 14-day-old rice seedlings (Co39), and at 5 days post incubation (dpi), only a few very small lesions were observed on rice leaves infected with the *ΔMonap1* mutant. Instead, more necrotic lesions were produced by Guy11 and *ΔMonap1/MoNAP1* (Figure 4C). These results suggested that *MoNAP1* is essential for *M. oryzae* pathogenicity. 

To explore the reasons for pathogenicity attenuation in the *ΔMonap1* mutant, appressorium-mediated penetration assay was performed using conidia of all tested strains on detached barley leaves, and expansion ability of the infected hyphae was observed in barley epidermis cell. We found that approximately 70% IH displayed type Ⅲ, 20% showed type Ⅱ, and 10% showed type Ⅰ at 24 hpi in WT and complemented strains *ΔMonap1/MoNAP1* (Figure 4D,E). However, in the *ΔMonap1* mutant, less 20% IH showed type Ⅲ, 40% showed type Ⅱ, and 20% showed type Ⅰ. These results suggested that *MoNAP1* is involved in infectious growth (Figure 4D,E). Taken together, these results suggested that *MoNAP1* is required for pathogenicity, and reduced invasive hyphae extension ability of *ΔMonap1* mutant strain may be the reason for the significant reduction in pathogenicity of *M. oryzae*.

### 2.5. ΔMonap1 Results in Accumulation of Reactive Oxygen Species (ROS) Produced by Host Cells

As mentioned in the above results, most infected hyphae of *ΔMonap1* had more difficulty in colonizing adjacent cells (Figure 4D,E). Therefore, we hypothesized that the ability to scavenge host reactive oxygen species was prevented in *ΔMonap1*. A 3,3′-diaminobenzidine (DAB) staining assay of the penetrated plant cells was performed. As shown in Figure 4F,G, approximately 70% of host cells penetrated by *ΔMonap1* mutant possessed ROS accumulation with reddish-brown color, while only about 30% of host cells infected with WT strain were stained by DAB, which suggested that the ability of *ΔMonap1* to eliminate host-produced ROS was decreased. Our result provided a demonstration that *MoNAP1* is involved in suppressing host oxidative burst.

### 2.6. MoNAP1 Is Required for Appressorium Formation and Utilization of Glycogen and Lipid Droplets

**Figure 5:**
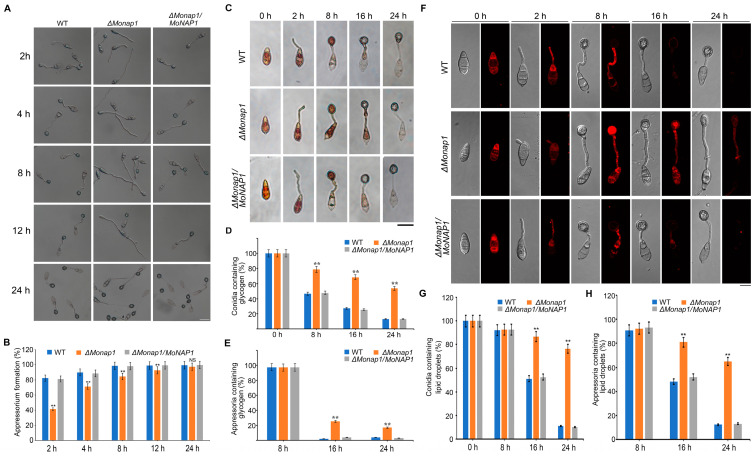
*MoNAP1* affects appressorium formation and utilization of glycogen. (**A**) Appressoria of tested strains induced in the artificial hydrophobic surface were observed under light microscope at 4, 6, 8, 12, and 24 h, respectively. Bar = 20 µm. (**B**) Statistical analysis of appressorium formation rate (%) of tested strains. For each strain, at least 100 conidia were observed, and the numbers of conidia that formed normal appressorium were counted. (**C**) Conidia of tested strains induced in the artificial hydrophobic surface and stained by KI/I_2_ solution were observed under light microscope at 0, 2, 8, 16, and 24 h, respectively. Bar = 20 µm. (**D**,**E**) Statistical analysis for proportion of conidia or appressoria containing glycogen. (**F**) Conidia and appressoria of tested strains induced in the artificial hydrophobic surface and stained by Nile red solution were observed under light microscope at 0, 2, 8, 16, and 24 h, respectively. Bar = 20 µm. (**G**,**H**) Statistical analysis for proportion of conidia or appressoria containing lipid droplets. For each strain, 100 conidia and appressoria were observed, and the numbers of stained conidia and appressoria were counted. Error bars represent standard deviation, and asterisks above the columns indicate significant differences between WT and *ΔMonap1* estimated by Student’s *t*-test (** *p* < 0.01).

The appressorium-mediated penetration is key for *M. oryzae* pathogenicity. To determine whether the *MoNAP1* was involved in the appressorium formation, we performed appressorium assay on hydrophobic surfaces. The appressorium formation rates of the wild-type strain Guy11, the *ΔMonap1* mutant, and the complementation strain *ΔMonap1/MoNAP1* were observed on the hydrophobic surfaces after 4, 6, 8, 12, and 24 hpi. We found that the appressorium formation rate was significantly delayed in the *Δmonap1* mutant after 4, 6, 8, and 12 hpi compared with Guy11 and the complementation strain *ΔMonap1/MoNAP1*, while this delay became indistinguishable after 24 h (Figure 5A,B).

The formation of appressorium is accompanied by degradation of glycogen and lipid droplets. Considering that the appressorium formation rate of *ΔMonap1* was decreased, we supposed that the translocation and degradation of nutriments were also delayed. Hence, we observed the cellular distribution of glycogen and lipid droplets at 0, 2, 8, 16, and 24 h, while glycogen and lipid droplets were stained with I_2_/KI and Nile red solution, respectively. In WT, the glycogen was rapidly consumed after 16 h and disappeared at 24 h, while in *ΔMonap1* mutant, the glycogen was utilized more slowly and was still noticed at 24 h (Figure 5C–E). Similarly, at 16 hpi, more than 80% of the *ΔMonap1* conidia contained lipid droplets; however, only about 40% of Guy11 conidia contained lipid droplets. Even at 24 hpi, lipid droplets could be observed in approximately 70% of the *ΔMonap1* appressoria, whereas only 15% of the Guy11 appressoria contained lipid droplets (Figure 5F,G). These results indicated that the deletion of *MoNAP1* affects appressorium formation and metabolism of nutrients, which is important for sufficient turgor pressure for host penetration.

### 2.7. MoNAP1 Is Involved in Responses to Multiple Stressors

**Figure 6:**
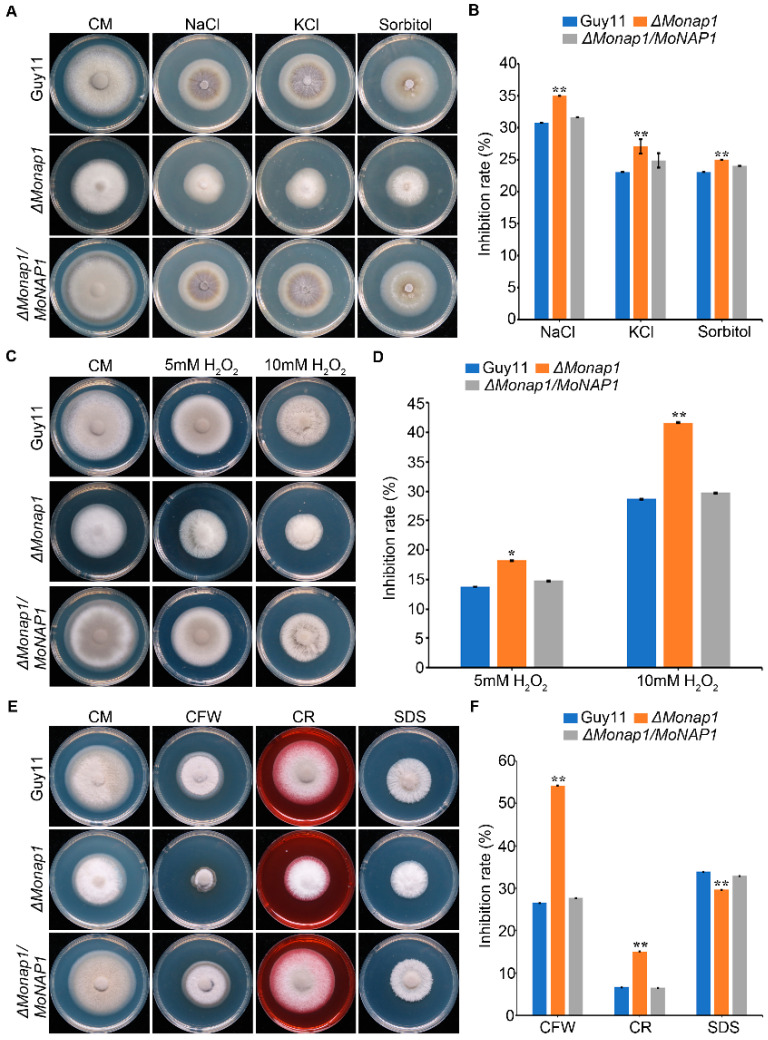
*ΔMoNAP1* mutant is sensitive to various stresses in *M. oryzae*. (**A**) Colony morphology of WT, *ΔMonap1*, and *ΔMonap1/MoNAP1* strains on CM plates supplemented with 1 M sorbitol, 0.7 M NaCl, and 0.6 M KCL_._ The colonies were measured and photographed at 7 dpi. (**B**) Statistical analysis of the relative inhibition rate (%) of the tested strains. (**C**) Colony morphology of WT, *ΔMonap1*, and *ΔMonap1/MoNAP1* strains on CM plates supplemented with 5mM H_2_O_2_ and 10 mM H_2_O_2._ The colonies were measured and photographed at 7 dpi. (**D**) Statistical analysis of the relative inhibition rate (%) of the tested strains. (**E**) Colony morphology of WT, *ΔMonap1*, and *ΔMonap1/MoNAP1* strains on CM plates supplemented with 200 µg/mL CFW, 600 µg/mL Congo red, and 0.004% SDS. The colonies were measured and photographed at 7 dpi. (**F**) Statistical analysis of the relative inhibition rate (%) of the tested strains. Relative inhibition rate = (the diameter of untreated strain − the diameter of treated strain)/(the diameter of untreated strain) × 100%. For each strain, three independent biological experiments with four replicates each time were carried out. Error bars represent standard deviation, and asterisks above the columns indicate significant differences between WT and *ΔMonap1* estimated by Student’s *t*-test (** *p* < 0.01).

In eukaryotes, a normal osmotic environment is essential for fungal growth and development. To determine the role of *MoNAP1* in hyperosmotic stress response, we tested the sensitivities of *ΔMonap1* mutant to different osmotic stress agents. The tested strains were cultured on CM plates containing 1 M sorbitol, 0.7 M NaCl, and 0.6 M KCl for 7 days. As shown in Figure 6A,B, *ΔMonap1* was more sensitive to all above-mentioned osmotic stresses compared with WT and *ΔMonap1/MoNAP1*, which indicates that *MoNAP1* plays an important role in responding to different osmotic stresses. In addition, the production of reactive oxygen species (ROS) is one of the most important defense responses in plants. According to the Figure 4F,G, we speculated that *MoNAP1* affects the sensitivity in responses to oxidative stresses. To confirm this, the relative inhibition rates of WT, *ΔMonap1*, and *ΔMonap1/MoNAP1* on CM with 5 mM and 10 mM H_2_O_2_ were examined. As expected, *ΔMonap1* was more sensitive to H_2_O_2_ and showed a more significant inhibition rate (Figure 6C,D). The cell wall integrity signaling pathway is important for blast fungus to resist external environmental stress. To test the sensitivity of *ΔMonap1* in response to cell wall integrity stress, WT, *ΔMonap1*, and *ΔMonap1/MoNAP1* were inoculated on CM plates supplemented with 200 µg/mL calcofluor white (CFW), 600 µg/mL Congo red (CR), and 0.004% sodium dodecyl sulfate (SDS). Figure 6E,F shows that *ΔMonap1* was more sensitive than the wild type and *ΔMonap1/MoNAP1* to CFW and CR but more resistant to SDS, indicating that the *MoNAP1* is involved in regulating the cell wall integrity pathway. Taken together, our results obtained from these assays suggested that the deletion of *MoNAP1* results in increased sensitivity to different stresses.

### 2.8. MoNap1 Interacts with Cell-Cycle-Related Proteins in M. oryzae

**Figure 7:**
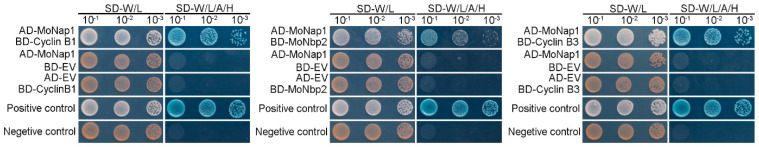
**Identification of MoNap1−interacted proteins in *M. oryzae***. Yeast two−hybrid (Y2H) assay. Yeast strains co-expressed prey and bait constructs were cultured on SD−Leu−Trp and SD−Ade−Leu−Trp−His plates supplemented with X−*α*−GAL, respectively. The positive control was the interaction between pGBKT7 −53 and pGADT7−T, and the negative control was the interaction between pGBKT7−Lam and pGADT7−T.

The Nap1 protein interacts with Nbp2 [25], Clb3 [8], Clb1 [8], Cdc28, Gin4 [21], Ats1 [32], and Cyc1 [8] in yeast. To determine whether MoNap1 interacts with Nbp2 (*MGG_03705*), Clb3 (*MGG_07065*), Clb1 (*MGG_03595*), Cdc28 (*MGG_01362*), Gin4 (*MGG_02810*), Ats1 (*MGG_05277*), and Cyc1 (*MGG_05646*) in *M. oryzae*, we performed a yeast two-hybrid (Y2H) assay. The result showed that only three proteins were shown to interact with MoNap1 in vitro (Figure 7 and Figure S2), including MoNbp2, MoClb3, and MoClb1. To further confirm their interaction in vivo, we performed a biomolecular fluorescent complimentary (BIFC) experiment. The results shown in Figure S3 indicate that MoNap1 interacts with MoNbp2, MoClb1, and MoClb3, respectively, in vivo. To further explore the connection between MoNap1 and its partner proteins, *ΔMonbp2* and *ΔMoclb3* mutant strains were generated (Figure S4). The deletion strain of *MoCLB1* was unsuccessful even when more than 300 knock-out transformants were examined, which suggests that *MoCLB1* is essential for the survival of *M. oryzae*. The pathogenicity of these mutant strains was analyzed, and results showed that the deletion of *MoNBP2* or *MoCLB3* has no effect on the pathogenicity of *M. oryzae* (Figure S4). 

**Figure 8:**
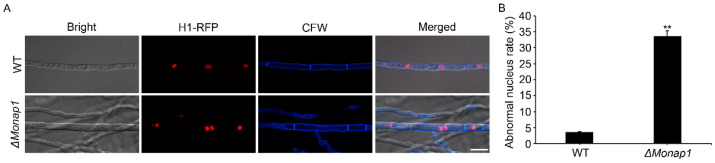
Subcellular localization of H1-RFP in WT and *ΔMonap1* mutant strains. (**A**) Subcellular localization of histone 1 in WT and *ΔMonap1* strains, respectively, in hyphae. H1-RFP was transformed into WT and *ΔMonap1* mutant. The cell wall was visualized by using calcofluorwhite (CFW). The co-localization was observed under NIKON laser scanning confocal microscope. Bar = 10 µm. (**B**) Statistical analysis for proportion of the abnormal quantity of nucleus in a single cell. For each strain, at least 100 vegetative hypha cells were observed and counted. Error bars represent standard deviation, and asterisks above the columns indicate significant differences between WT and *ΔMonap1* estimated by Student’s *t*-test (** *p* < 0.01).

Previous studies reported that deletion of *NAP1* results in the unnormal function of B-type cyclin Clb2 and causes prolonged mitotic delays in *S. cerevisiae* [8,14]. To investigate if *NAP1* affects cell cycle progression in *M. oryzae*, we transferred H1-RFP plasmid into WT and *ΔMonap1* mutant and then observed the localization of H1 during the vegetative hyphae stage. As shown in Figure 8A,B, there was only one nucleus in most WT cells but two or more cell nucleuses in more than 30% of the *ΔMonap1* cells. Our result showed that loss of *MoNAP1* significantly causes a disordered cell cycle in *M. oryzae*.

### 2.9. Transcriptional Analysis for Identification of Differentially Expressed Genes (DEGs) in a Comparison of the ΔMonap1 and Guy11

**Figure 9:**
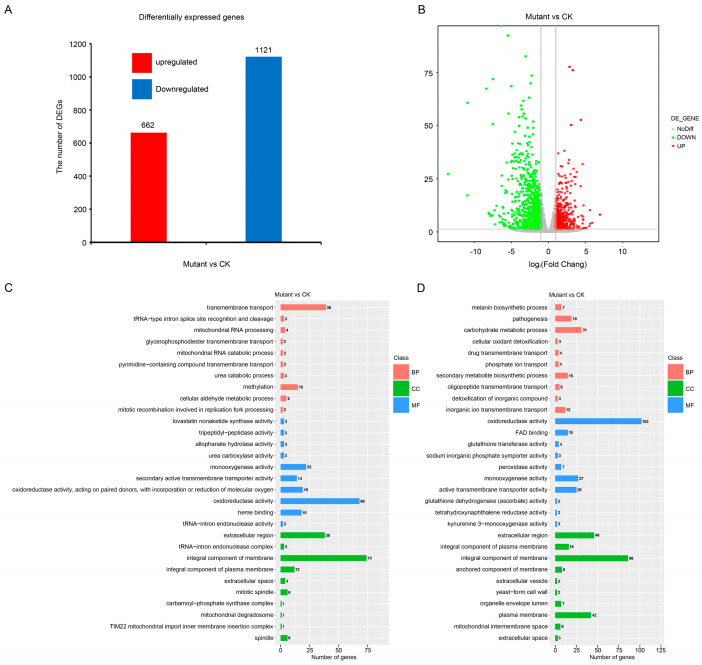
Transcriptional analysis for identification of differentially expressed genes (DEGs) in a comparison of the Guy11 and *ΔMonap1*. (**A**) Differentially expressed genes in WT and *ΔMonap1* mutant strains from RNAseq data. (**B**) Expression fold changes of genes in *ΔMonap1* compared with WT (|log_2_FoldChange| values > 1, Padj < 0.05). (**C**) Gene ontology (GO) enrichment analysis of the genes up-regulated. (**D**) Gene ontology (GO) enrichment analysis enrichment analysis of the genes down-regulated.

To identify the potential target genes regulated by *MoNAP1*, we performed transcriptome analysis between Guy11 and *ΔMonap1* mycelia. A total of 1783 DEGs were identified between Guy11 and the *ΔMonap1* mutant using the standard criterion of |log_2_FoldChange|values > 1 and Padj < 0.05, which include 1121 down-regulated and 662 up-regulated genes (Figure 9A,B). Gene ontology (GO) enrichment analysis of the genes up-regulated and down-regulated showed that the biological processes of transmembrane transport, carbohydrate metabolic process, pathogenesis, secondary metabolite biosynthetic process, and melanin biosynthetic process were enriched. For cellular components, integral component of membrane, extracellular region, plasma membrane, integral component of plasma membrane, mitotic spindle, and spindle were enriched (Figure 9C,D and Figure S5). We also selected two pathogenesis-related genes to perform qRT-PCR to further identify the authenticity of RNA-seq results, and the results in Figure S6 suggest that the expression level of *MoZFP6* and *MoDIT2* were severely decreased compared to WT (Table S2). These results obtained from GO enrichment analysis further confirmed that *MoNAP1* is involved in utilization of glycogen, in the cell cycle, and in response to different stresses.

In addition, a total of 1783 genes were mapped to Kyoto Encyclopedia of Genes and Genomes (KEGG) pathway, and the top 30 significantly enriched (Padj < 0.05) pathways are shown in Figure S7, which include amino acid metabolism (beta-alanine metabolism, tryptophan metabolism, phenylalanine metabolism, glutathione metabolism, and tyrosine metabolism), lipid metabolism (fatty acid degradation), betalain biosynthesis, and metabolism of xenobiotics by cytochrome P450. The KEGG pathway enrichment analysis indicated that *MoNAP1* is important for amino acid metabolism and lipid metabolism in *M. oryzae*. 

## 3. Discussion

Nucleosome assembly protein 1 (Nap1) plays important roles in diverse biological processes [2]. However, the biological functions of *MoNAP1* in filamentous fungus have rarely been reported. In this study, we identified the nucleosome assembly protein MoNap1 in *M. oryzae*. We found MoNap1 is essential for vegetative growth, asexual reproduction, appressorium formation, and pathogenicity in *M. oryzae*. Moreover, we identified and verified three proteins that interact with MoNap1. Additionally, transcriptome data suggested important roles of *MoNAP1* in pathogenicity and pigmentation biosynthesis-related pathways.

In the yeast *S. cerevisiae*, deletion of *NAP1* causes no phenotypes or very limited phenotypes [8,22]. Our results showed that deletion of *MoNAP1* reduced the growth rate of colony and spore quantity in *M. oryzae*. The colonies of the mutant appear whiter than the WT and complemented strains. The expression levels of *MoALB1* and *MoBUF1* that regulate the formation of melanin were tested. The result indicated that *MoNAP1* is involved in the pathway related to melanin formation. Further experiments showed that the deletion of *MoNAP1* caused a significant reduction in pathogenicity on different plants and restricted the extension of invasive hyphae in plant cells. 

Considering that infection sites of the *ΔMonap1* mutant are much fewer than in Guy11 and *ΔMonap1/MoNAP1*, we speculated that the appressorium formation rate of *ΔMonap1* was significantly reduced compared to Guy11 and *ΔMonap1/MoNAP1*. As expected, the appressorium formation rate of *ΔMonap1* was approximately 84% at 8 h, which confirmed our hypothesis. In addition, rapid degradation of glycogen and lipid droplets occurred during conidia germination, followed by accumulation in incipient appressoria and dissolution before turgor generation. According to the above results, we confirmed the slower degradation of glycogen lipid droplets. As shown in our results, the degradation of nutriments was delayed in *ΔMonap1* mutant compared to WT and complemented strains, which confirmed our hypothesis. 

Stress-signaling pathways are evolutionarily conserved and play an important role in the maintenance of homeostasis. These pathways are also critical for adaptation to new cellular environments [33]. The cell wall is a highly dynamic structure that is responsible for protecting the cell from rapid changes in external osmotic potential. The wall is also critical for cell expansion during growth and morphogenesis [34]. Here, we explored the roles of *MoNAP1* in response to cell-wall-integrity stress. As result, the *ΔMonap1* mutant is more sensitive than WT and complemented strains in CFW and CR but less in SDS, which suggests *MoNAP1* is involved in cell wall integrity. The ability of cells to adapt to hyperosmotic stress involves early responses in which ions move across cell membranes and late responses characterized by increased synthesis of either membrane transporters or enzymes involved in their synthesis. The goal of these responses is to return the cell to its normal size and maintain cellular homeostasis [35]. To test the sensitivity of *ΔMonap1* to hyperosmotic stress, *ΔMonap1*, Guy11, and *ΔMonap1/MoNAP1* were treated with hyperosmotic stress factors. Our results showed that *ΔMonap*1 is more sensitive to these factors, which indicated the importance of the adaptation of *MoNAP1* to hypertonic stresses in *M. oryzae*. During plant infection, both pathogenic and beneficial fungi experience a host-derived oxidative burst of reactive oxygen species (ROS) [36]. This burst is part of the plant’s innate immune response and must be subdued in order to avoid triggering more robust plant defenses. Besides overcoming plant-derived ROS, fungi must also overcome ROS produced as a byproduct of aerobic respiration [37]. Given that, we detected the sensitivity of *ΔMonap1* to H_2_O_2_, and our data showed that the *ΔMonap1* mutant was more sensitive to H_2_O_2_. From these findings, we concluded that *MoNAP1* plays an indispensable role in regulating oxidative stress.

Nap1 is generally localized in the cytoplasm except that subcellular localization of *Drosophila melanogaster* Nap1 is dynamically regulated between the cytoplasm and nucleus during early development [6]. In the yeast *Saccharomyces cerevisiae*, yNap1 is also a nucleocytoplasmic shuttling protein [38]. Here, we verified that MoNap1 was located in cytoplasm but not nucleus in both conidia, appressorium, vegetative mycelia, and infection mycelia. In yeast, deletion of the nuclear export sequence (NES) makes yNap1 locate to the cell nucleus [38]. However, when NES was deleted in *MoNAP1* of *M. oryzae*, the localization of MoNap1 appears to be impervious [39]. The reason for this contradiction remains to be investigated.

Nbp2p is a Nap1-binding protein in *Saccharomyces cerevisiae* identified by its interaction with Nap1 by a two-hybrid assay [25]. Nbp2 regulates mitotic cell growth at high temperature and the cell-wall-integrity pathway [25]. Furthermore, the interaction between B-type cyclins and Nap1 proteins is highly conserved during evolution [8]. In this study, we identified that MoNap1 also interacts with MoNbp2, MoClb1, and MoClb3 in *M. oryzae*. Deletion of MoNbp2 and MoClb1 did not affect the pathogenicity in *M. oryzae*. In addition, our failure to obtain an *MoCLB3* deletion mutant implies that *MoCLB3* is likely to be an essential gene. Furthermore, transcriptome data suggested that deletion of *MoNAP1* significantly reduced the expression level of many pathogenesis-related and melanization-related genes. In conclusion, our study provides new proof that *MoNAP1* is required for growth, asexual reproduction, and pathogenicity, and *MoNAP1* regulates the appressorium formation rate, melanization, and cell cycle. Meanwhile, we identified three MoNap1-interacting proteins, which may be of benefit in further exploring the function of *MoNAP1* in *M. oryzae*.

## 4. Materials and Methods

### 4.1. Strains and Culture Conditions 

The *M. oryzae* WT strain Guy11 and all strains obtained in this study were cultured on complete medium (CM) at 28 °C. To extract DNA, RNA, and protein, mycelia of all tested strain were inoculated in liquid CM medium for 48 h. For growth assay, equal-sized plugs of each strain tested were cultivated on CM agar at 28 °C for 7 days, and diameter of colony was measured. For conidiation, conidia from colonies cultured on RDC medium at 28 °C for 5 days in dark followed by 3 days of continuous illumination under fluorescent light were harvested for experiment. For appressorium formation assay, a suspension of conidia of all tested strains was cultured on hydrophobic surface to induce formation of appressorium at 28 °C in dark. The rate of appressorium formation was counted at 4 h, 6 h, 8 h, 12 h, and 24 h, respectively, and the images were obtained with inverted fluorescence microscopy (Nikon, Tokyo, Japan).

For sensitivity assays, strains were inoculated on CM plates supplemented with different stress agents (1 M sorbitol, 0.7 M NaCl, 0.6 M KCL, 200 µg/mL CFW, 600 µg/mL Congo red, 0.004% SDS, and 10 mM H_2_O_2_) at 28 °C in dark. The colony diameters were measured after 7 days post inoculation (dpi). All treatments were performed in three independent biological experiments with three replicates. The significance analysis was performed using *t*-test formula in Excel software. 

### 4.2. Plasmid Constructs and Genetic Transformation

To generate *ΔMonap1* deletion mutants, a high-throughput target-gene deletion system was used to construct gene deletion vector [40]. The 1.2 kb upstream fragment (UF) and 1.2 kb downstream fragment (DF) of the targeted ORF were, respectively, amplified from the *M. oryzae* genome using primers pairs listed in Table S1. The hygromycin-resistance cassette (*HPH*) sequence was amplified from the plasmid pFGL821 using pairs listed in Table S1. All of these fragments (UF, DF, and *HPH*) were cloned into vector pKO1B with the *Xba* I*/Hin*d III-linearized by one-step cloning kit (Vazyme Biotech Co. C113, Nanjing, China). After PCR and sequencing verification, the correct plasmid was transferred into *M. oryzae* WT strain Guy11 through *Agrobacterium tumefaciens*-mediated transformation (ATMT). The transformants were screened using CM medium containing 250 µg/mL hygromycin B and further verified by PCR and RT-PCR using pairs listed in Table S1. The positive transformants were confirmed by Southern blot analysis. Four independent mutants with similar phenotype were obtained, and one of all mutants was used for further analysis. 

For complementation of the mutants *ΔMonap1* and subcellular localization of MoNap1, the complement fragments containing the native promoter and ORF without stop codon were amplified by PCR using pairs listed in Table S1 and inserted into pYF11 as previously described [41]. The construct was transformed into deletion mutant and WT through protoplast transformation, respectively. The transformants were screened using CM medium containing 60 µg/mL bleomycin and further verified by RT-PCR using pairs listed in Table S1. To generate a nucleus marker construct MoH2B-mCherry, fragments including the *MoH2B* without stop codon and its native promoter were amplified from Guy11 genomic DNA by PCR using pairs listed in Table S1, and the mCherry fragment was amplified using pairs listed in Table S1. All of these fragments (MoH2B and mCherry) were cloned into vector pKNT with the *Xho* Ⅰ/*Bam*H Ⅰ-linearized by Clonexpress Multis One-Step Cloning Kit (Vazyme Biotech Co. C113, China). After PCR and sequencing verification, the correct plasmid MoH2B-mCherry was transferred into was transformed into strain-expressed *MoNAP1-GFP* in background using PEG-mediated method. The transformants were screened using CM medium containing 200 mg/mL G418 or confirmed by the presence of mCherry signals. 

### 4.3. Pathogenicity and Infection Penetration Assays

To determine the virulence of all tested strains, we performed pathogenicity and infection assays on 7-day-old barley (Golden Promise) and 14-day-old rice seedlings (CO39). First, mycelial blocks or spore suspensions of all tested strains were inoculated onto intact and injured barley leaves and kept in the dark incubator for 24 h at 28 °C with 90% humidity, followed by 12 h light:12 h dark photoperiod for 5 days. Then, the disease spots were observed and imaged. In addition, spore suspension (5 × 10^4^ conidia/mL) collected from all tested strains were sprayed onto 14-day-old rice seedlings (CO39). The disease spots were observed and imaged after 5 days. Furthermore, for infection penetration, 10 µL spore suspension per drop was inoculated onto the back of the barley and then cultured at 28 °C for 24 h under dark conditions. After that, the barley epidermis was observed by using inverted fluorescence microscope (Nikon, Japan). Each assay was repeated three times. 

### 4.4. Nucleic Acid Manipulation, Sothern Blot Analysis, and RT-PCR and Real-Time qRT-PCR Analysis

Genomic DNA of all tested strains were extracted from the mycelia following the SDS-CTAB method. The resulting genomic DNA was used for PCR or Sothern blot analysis. Sothern blot assay was performed as in the previous study [41]. The flank sequence of target gene was used as the specific probe, which was labelled with the DIG High Prime. Hybridization and detection were performed according to manufacturer’s instructions (Roche Applied Science, Penzberg, Germany). Total RNA was isolated from fresh mycelia using RNeasy Mini Kit (Qiagen, 74104, Hilden, Germany). Then, 5 mg of total RNA of each sample returned was reverse-transcribed to cDNA using HiScript II 1st Strand cDNA Synthesis Kit (+gDNA wiper) (Vazyme, China). RT-PCR was performed to confirm the deletion and the complementation of targeted gene with gene-specific primers (Table S1). qRT-PCR was performed with 2×RealStar Green Fast Mixture (Genstar, San Francisco, CA, USA) using CFX Connect Real-Time PCR Detection System. The *ACTIN* gene (*MGG_03982*) was used as an endogenous reference. The experiment was repeated three times with three replicates each time. All primers used in these assays are listed in Table S1.

### 4.5. Glycogen and Lipid Droplet Straining

Glycogen and lipid droplet staining was observed at 0 h, 2h, 8 h, 16 h, and 24 h using KI/I_2_ solution and Nile red solution, respectively. A laser scanning confocal microscope (Nikon, Tokyo, Japan) was used to image red fluorescence.

### 4.6. Subcellular Localization 

The transformant co-expressed MoH2B-mCherry and MoNap1-GFP was used to observed subcellular localization at different stages using a Nikon TIE system (Nikon, Japan). The assay was repeated three times.

### 4.7. Yeast Two-Hybrid (Y2H) Assay

The full-length cDNA of *MoNAP1* was amplified and cloned into pGADT7 as the prey construct pGADT7-MoNap1, and the cDNA of *MoNBP2* (*MGG_03705*), *MoCLB3* (*MGG_07065*), *MoCLB1* (*MGG_03595*), *MoCDC28* (*MGG_01362*), *MoGIN4* (*MGG_02810*), *MoATS* (*MGG_05277*), and *MoCYC1* (*MGG_05646*) were amplified and cloned into pGBKT7 as the bait construct, respectively. Corresponding primers are listed in Table S1. Both the prey construct and the bait construct were co-transformed into yeast strain Y2HGold based on the manufacturer’s instructions (Matchmaker Gold Yeast Two-Hybrid System).

### 4.8. Bimolecular Fluorescence Complementation (BIFC) Assay

The plasmid pKD2-YFP^CTF^ (hygromycin B resistance) was digested with BamHI and then ligated with MoNAP1 fragment amplified with a primer pair (MoNAP1-YFPCF/MoNAP1-YFPCR). The plasmid pKD5-YFP^NTF^ (sulfonylurea resistance) was digested with XbaI and the ligated with MoNBP2, MoCLB1, and MoCLB3 fragments amplified with three primer pairs, respectively (MoNBP2-YFPNF/MoNBP2-YFPNR, MoCLB1-YFPNF/MoCLB1-YFPNR, and MoCLB3-YFPNF/MoCLB3-YFPNR). Afterwards, pKD2-MoNAP1-YFP^CTF^ was co-transformed into Guy11 with pKD5-MoNBP2-YFP^NTF^, pKD5-MoCLB1-YFP^NTF^, and pKD5-MoCLB3-YFP^NTF^ via ATMT, respectively, and the transformants were screened in CM supplemented with hygromycin B and sulfonylurea. The fluorescence signals were observed under laser scanning confocal microscope (NIKON, Japan).

### 4.9. Transcriptome Analysis

The wild-type strain Guy11 and *ΔMonap1* mutant were incubated in CM liquid medium at 28 °C for 48 h. Three replicates were performed for each strain. The mycelium pellets were collected and snap-frozen with liquid nitrogen. Total RNA from the wild-type strain Guy11 and *ΔMonap1* mutant was extracted using Qiagen RNAeasy Mini kit (Qiagen, 74104) according to the manufacture’s protocol. The integrity of RNA was evaluated using the RNA nano 6000 assay kit (Agilent Technologies, Santa Clara, CA, USA) of Agilent 2100 Bioanalyzer. The RNA with poly-A was enriched by TIANSeq mRNA Capture Kit (TIANGEN). Then, using the captured RNA as the starting sample, TIANSeq Fast RNA Library Kit (Illumina, San Diego, CA, USA) was used to construct the transcriptome sequencing libraries, and sequencing was performed on an Illumina Novaseq platform by (Beijing Tangtang Tianxia Biotechnology Co., Ltd., Beijing, China).

Differentially expressed genes were analyzed by the DESeq2 package, and expression with log2|FC| > 1 with <0.05 padj values was defined as DEGs [42]. Ontology enrichment analysis was performed using the topGO R package, and p-adjust values < 0.05 were considered significantly enriched by DEGs [43]. KEGG enrichment analysis was performed using clusterProfiler R package to test the statistical enrichment of differential expression genes in KEGG pathways [44].

## Data Availability

The data presented in this study are available in this published article or its Supplementary Materials.

## Supplementary Materials

The following supporting information can be downloaded at: https://www.mdpi.com/article/10.3390/jof9010050/s1, Figure S1: Targeted gene deletion of MoNAP1 in *M. oryzae*; Figure S2: MoNap1 has no interaction with MoCdc28, MoGin4, MoAts1 and MoCyc1 in *M. oryzae*; Figure S3: BiFC results between MoNap1-YFP^CTF^ and YFP^NTF^-MoNbp2, YFP^NTF^-MoClb1 or YFP^NTF^-MoClb3; Figure S4: Knockout and phenotype analysis of MoNap1-interacted proteins; Figure S5: Gene Ontology terms of biological processes, cellular components, and molecular functions enriched in wild-type strain Guy11 and ΔMonap1 mutant; Figure S6: Expression level of *MoZFP6* and *MoDIT2* in WT and ΔMonap1 mutant; Table S1: Primers used in this study; Table S2: Differentially expressed genes related to melanin biosynthesis and pathogenesis in *ΔMonap1* mutant.
