# Supplementary material for: MoNap1, a Nucleosome Assemble Protein 1, Regulates Growth, Development, and Pathogenicity in Magnaporthe oryzae
Source: J Fungi (Basel). 2022 Dec 28;9(1):50. doi: 10.3390/jof9010050 (PMC9862126; doi:10.3390/jof9010050)
Supplement: Supplementary file 1 [file jof-09-00050-s001.zip › jof-2089421-SI/Fig.S3.pdf]

Figure S3

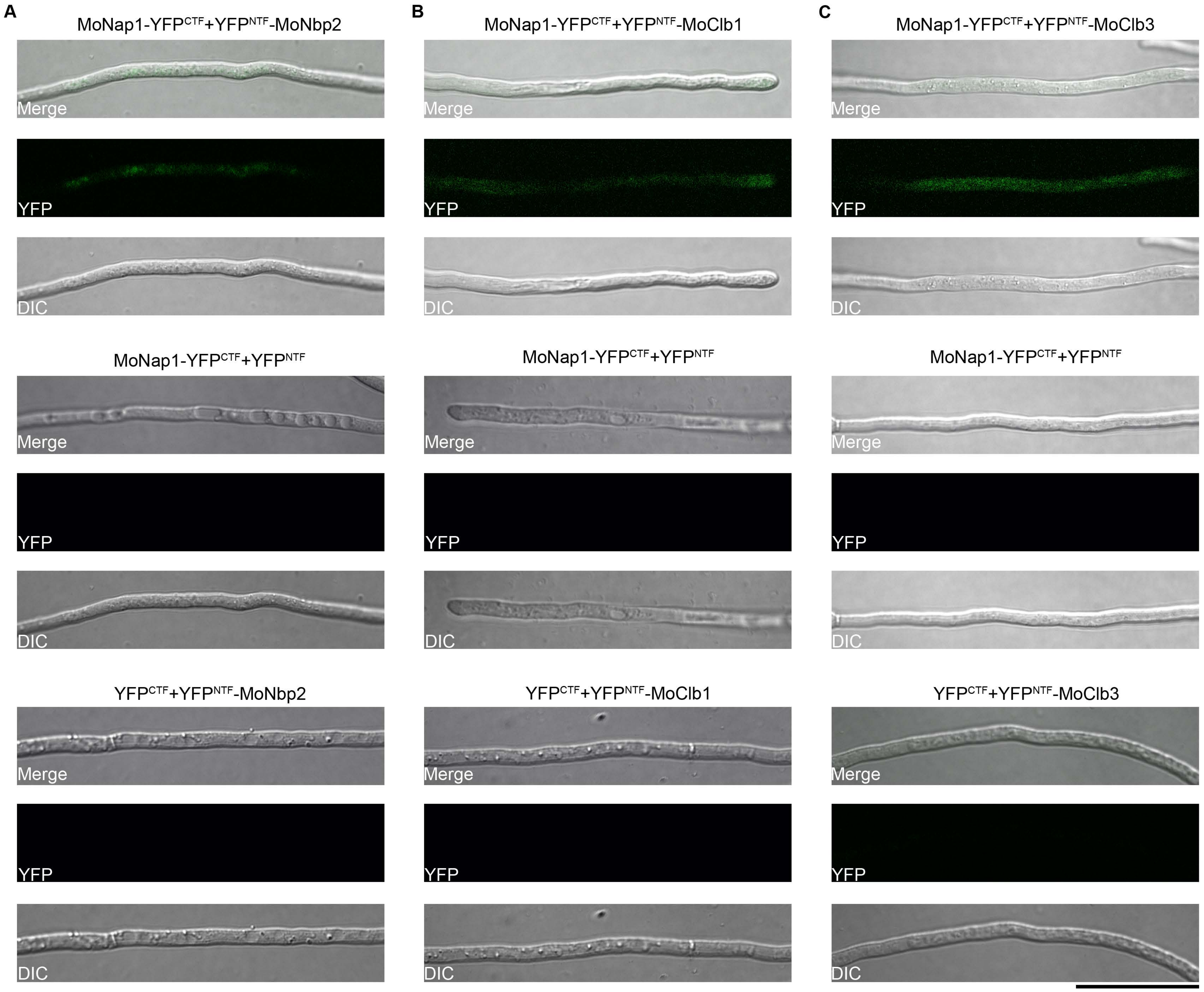
A
MoNap1-YFPCTF+YFPNTF-MoNbp2
Merge
YFP
DIC
MoNap1-YFPCTF+YFPNTF
Merge
YFP
DIC
YFPCTF+YFPNTF-MoNbp2
Merge
YFP
DIC
B
MoNap1-YFPCTF+YFPNTF-MoClb1
Merge
YFP
DIC
MoNap1-YFPCTF+YFPNTF
Merge
YFP
DIC
YFPCTF+YFPNTF-MoClb1
Merge
YFP
DIC
C
MoNap1-YFPCTF+YFPNTF-MoClb3
Merge
YFP
DIC
MoNap1-YFPCTF+YFPNTF
Merge
YFP
DIC
YFPCTF+YFPNTF-MoClb3
Merge
YFP
DIC
