# Supplementary material for: MoNap1, a Nucleosome Assemble Protein 1, Regulates Growth, Development, and Pathogenicity in Magnaporthe oryzae
Source: J Fungi (Basel). 2022 Dec 28;9(1):50. doi: 10.3390/jof9010050 (PMC9862126; doi:10.3390/jof9010050)
Supplement: Supplementary file 1 [file jof-09-00050-s001.zip › jof-2089421-SI/Supplementary material.pdf]

Supplementary material

MoNap1, a nucleosome assemble protein 1, regulates growth, development and pathogenicity in *Magnaporthe oryzae*

Shulin Zhang[1,2#*], Yu Wang[1,2#], Xinyue Cui[1,2#], Jinmei Hu[1,2], Xiaoru Kang[1,2], Yuyan Liu[1,2], Yuemin Pan[1,2*]

1. Department of Plant Pathology, College of Plant Protection, Anhui Agricultural University, Hefei, Peoples' Republic of China 230036.

2. Anhui Province Key Laboratory of Crop Integrated Pest Management, Anhui Agricultural University, Hefei, Peoples' Republic of China 230036.

# These authors contributed equally to this work

* Address correspondence to SL Zhang (zhangsl80h@ahau.edu.cn) and YM Pan (panyuemin2008@163.com)

This PDF file includes

Figure and Table

Figure S1 Targeted gene deletion of *MoNAP1* in *M.oryzae*. (A) Schematic presentation of targeted deletion of *MoNAP1* using homologous recombination method. **(B)** PCR verification of knockout transformants. **(C)** Southern blot assay showing the successful replacement of *MoNAP1* by a single insertion of hygromycin fragment. **(D)** RT-RCP verification of mutant and complemented strains.

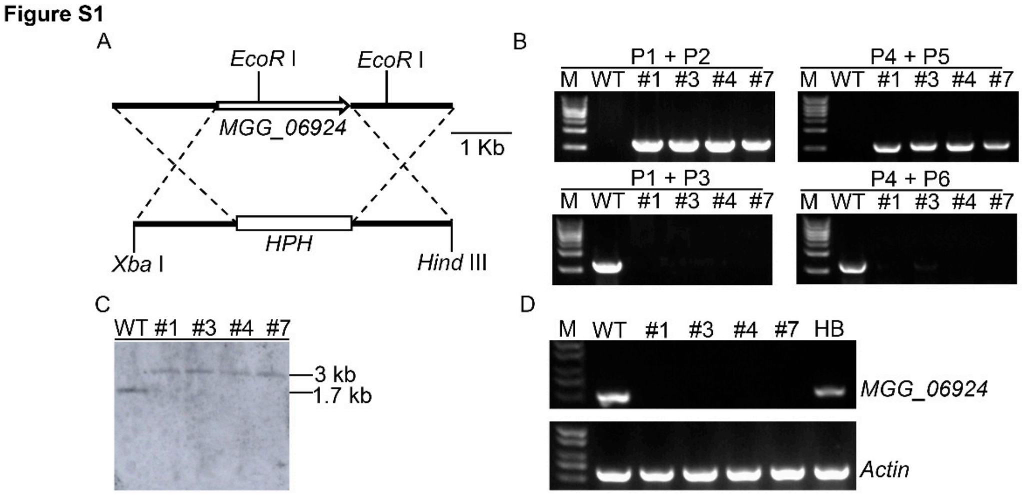

Figure S2 MoNap1 has no interaction with MoCdc28, MoGin4, MoAts1 and MoCyc1 in *M.oryzae*.

Yeast two hybrid (Y2H) assay. Yeast strains co-expressing prey and bait constructs were cultured on SD-Leu-Trp and SD-Ade-Leu-Trp-His plates supplemented with X-α-GAL respectively.

Figure S2

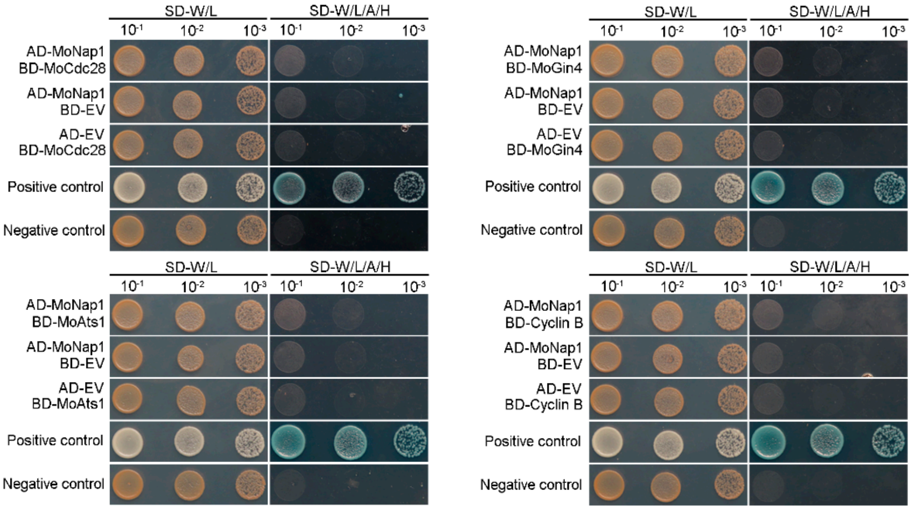

Figure S3. BiFC results between MoNap1-YFP^{CTF} and YFP^{NTF}-MoNbp2, YFP^{NTF}-MoClb1 or YFP^{NTF}-MoClb3. YFP signals were observed in vegetative hyphae under laser scanning confocal microscope (NIKON, Japan). The negative control transformants harbor MoNap1-YFP^{CTF} and YFP^{NTF}, YFP^{CTF} and YFP^{NTF}-MoNbp2, YFP^{CTF} and YFP^{NTF}-MoClb1, YFP^{CTF} and YFP^{NTF}-MoClb3. Bar=25 μm.

Figure S3

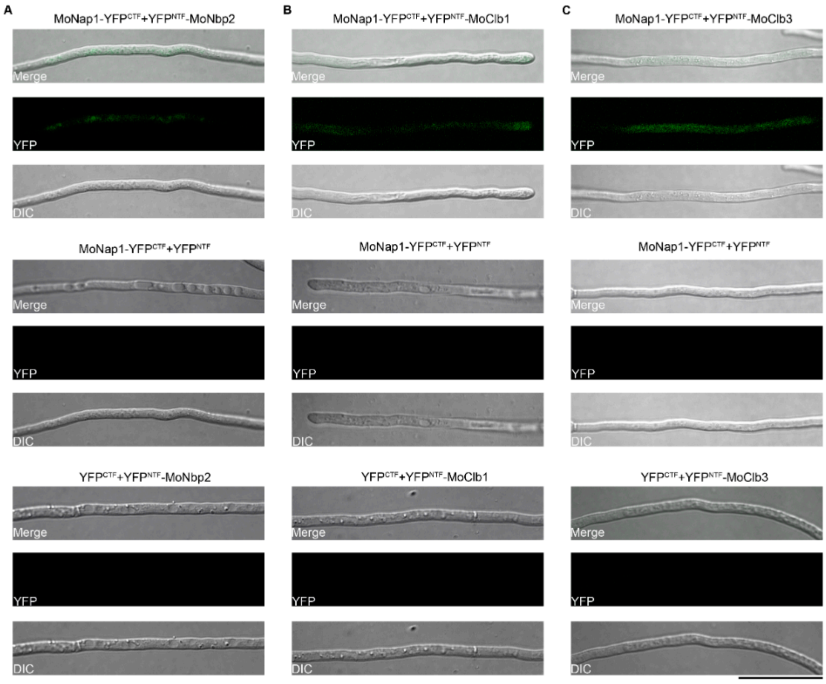

Figure S4 Knockout and phenotype analysis of MoNap1-interacted proteins. (A) and (B) Schematic presentation of targeted deletion of *MoNBP2* and *MoCLB3* using homologous recombination method. (C) and (D) Gene knockout verification of *ΔMonbp2* and *ΔMoclb3* mutants by Southern blot analysis. (E) Colony morphology of WT, *ΔMonbp2* and *ΔMoclb3* mutant strains on CM were observed and photographed after 7 days at 28 °C. (F) The colony diameters were measured and subjected to statistical analysis. For each strain, three independent biological experiments with four replicates each time were carried out. Error bars represent standard deviation, and asterisks above the columns indicate significant differences between WT and *ΔMonbp2,* WT and *ΔMoclb3* estimated by Student's t test (***P*<0.01). (G) Virulence of WT, *ΔMonbp2* and *ΔMoclb3* mutant strains performed on detached barley leaves. Lesions formed on barley leaves inoculated with mycelial blocks of WT, *ΔMonbp2* and *ΔMoclb3* and observed at 5dpi.

Figure S4

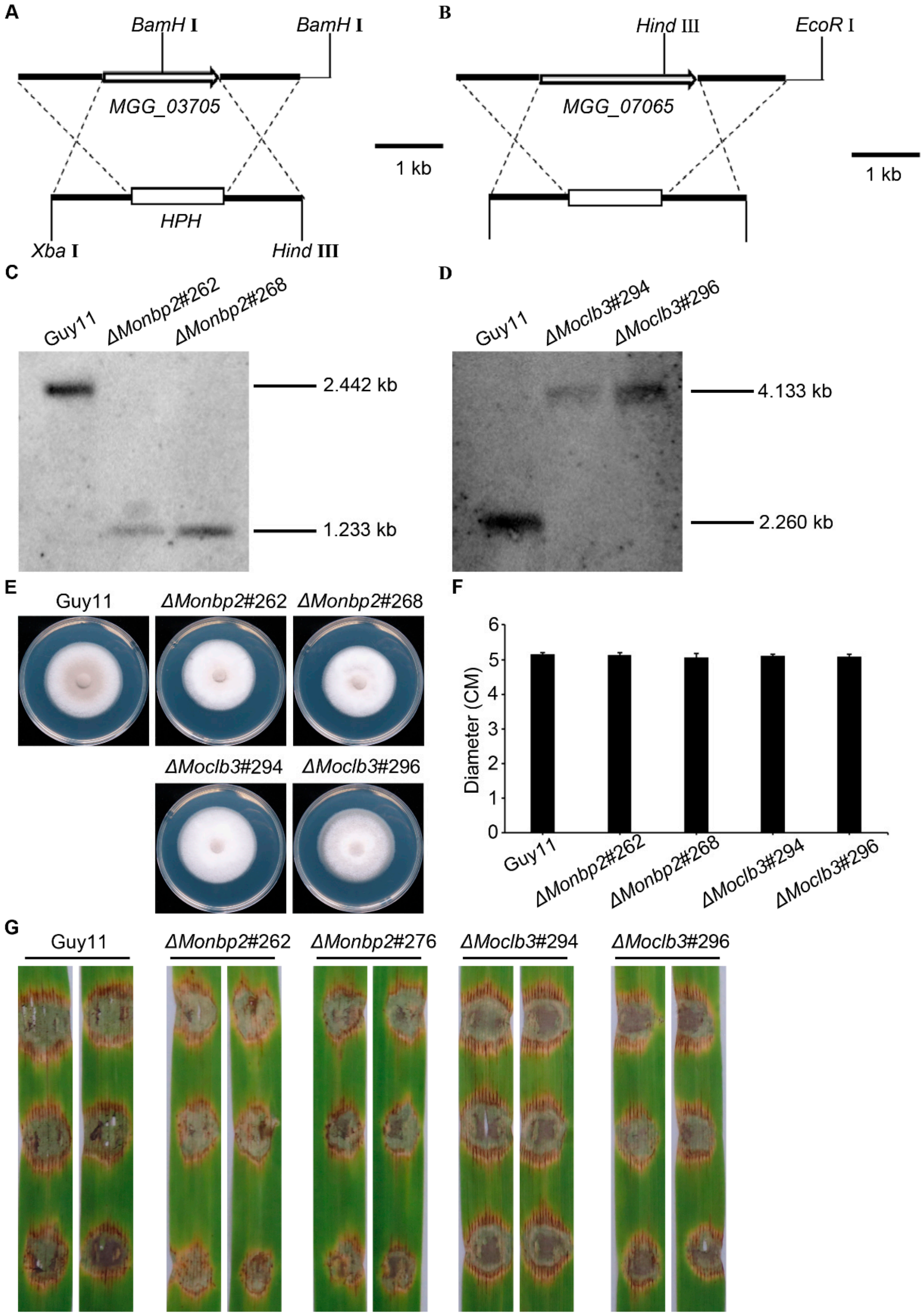
A
BamH I
BamH I
MGG_03705
1 kb
HPH
Xba I
Hind III
B
Hind III
EcoR I
MGG_07065
1 kb
C
Guy11
ΔMonbp2#262
ΔMonbp2#268
2.442 kb
1.233 kb
D
Guy11
ΔMoclb3#294
ΔMoclb3#296
4.133 kb
2.260 kb
E
Guy11
ΔMonbp2#262
ΔMonbp2#268
ΔMoclb3#294
ΔMoclb3#296
F
Diameter (CM)
0
1
2
3
4
5
6
Guy11
ΔMonbp2#262
ΔMonbp2#268
ΔMoclb3#294
ΔMoclb3#296
G
Guy11
ΔMonbp2#262
ΔMonbp2#276
ΔMoclb3#294
ΔMoclb3#296

Figure S5. Gene Ontology terms of biological processes, cellular components, and molecular functions enriched in wild-type strain Guy11 and *ΔMonap1* mutant. (A) Gene Ontology (GO) analysis of up-regulated genes ($P<0.05$). (B) Gene Ontology (GO) analysis of down-regulated genes ($P<0.05$).

Figure S5

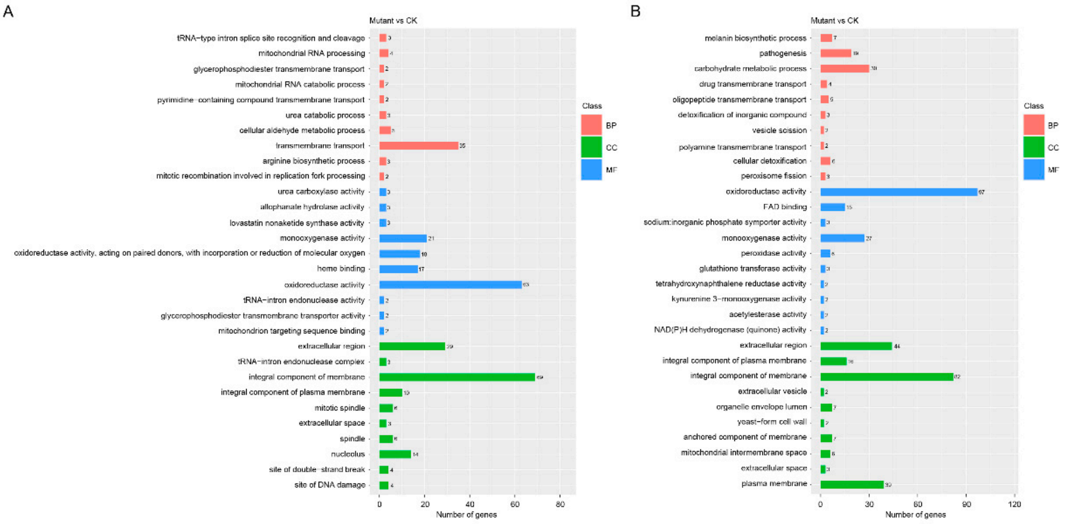

Figure S6. Expression level of *MoZFP6* and *MoDIT2* in WT and *ΔMonap1* mutant. The expression level of different genes in WT was set as 1.

Figure S6

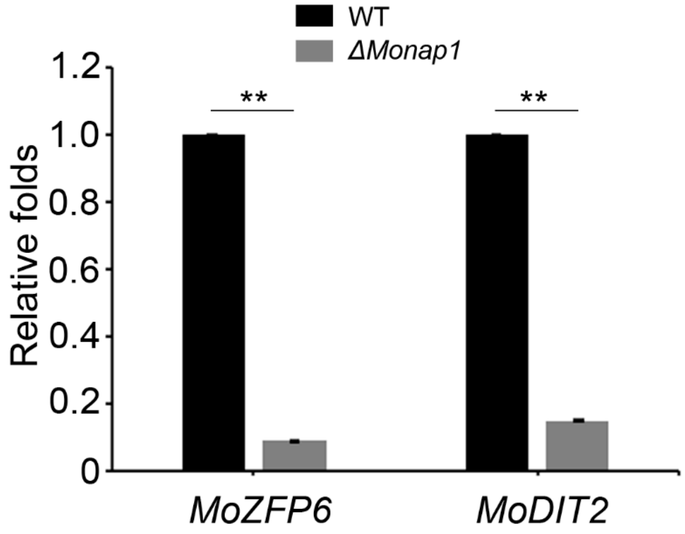

Figure S7 Kyoto Encyclopedia of Genes and Genomes (KEGG) pathway enrichment analysis of the DEGs. (A) Kyoto Encyclopedia of Genes and Genomes (KEGG) pathway enrichment analysis of the genes up-regulated. **(B)** Kyoto Encyclopedia of Genes and Genomes (KEGG) pathway enrichment analysis of the genes down-regulated.

Figure S7

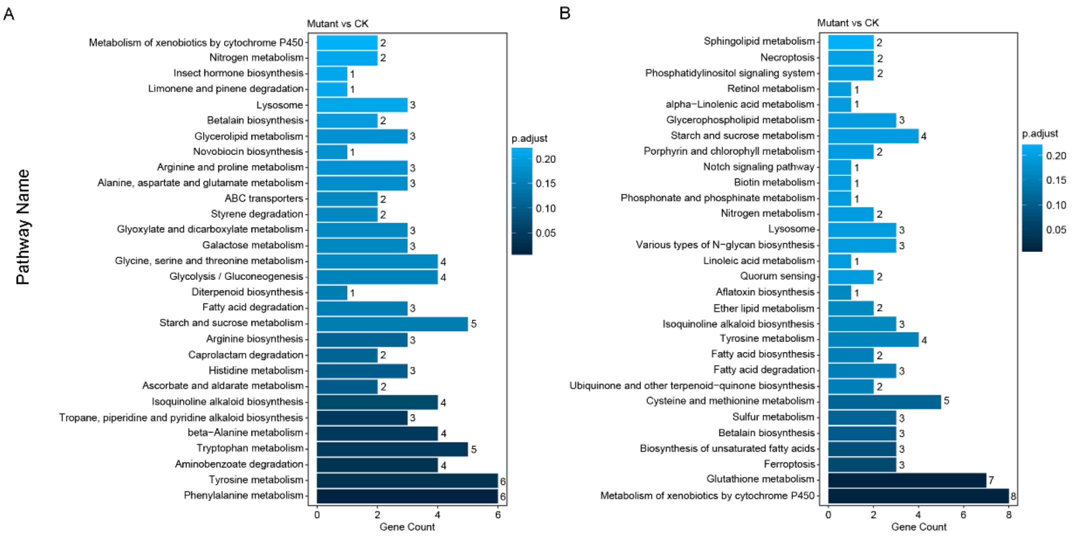

Table S1. Primers used in this study

Table S2. Differentially expressed genes related to melanin biosynthesis and pathogenesis in *ΔMonap1* mutant using $|log_2FoldChange| > 1.5$ and $P < 0.05$.
