# Supplementary material for: MoNap1, a Nucleosome Assemble Protein 1, Regulates Growth, Development, and Pathogenicity in Magnaporthe oryzae
Source: J Fungi (Basel). 2022 Dec 28;9(1):50. doi: 10.3390/jof9010050 (PMC9862126; doi:10.3390/jof9010050)
Supplement: Supplementary file 1 [file jof-09-00050-s001.zip › jof-2089421-SI/Table S1. Primers used in this study.pdf]

Table S1. Primers used in this study

Primer Name	Sequences (5'-3')	Remarks
**MoNap1_upF**	GGTACCCGGGGATCCTCTAGACTACTCCATACTTTGGGCGGGTTGGTA	Amplify 5'*MoNAP1* upstream fragment
**MoNap1_upR**	TTCATTGTTGACCTCCACTACGGTCCTACTGCAGATGACCTCTTCTCT	Amplify 5'*MoNAP1* upstream fragment
**MoNap1_downF**	GCAAAGGAATAGAGTAGATATAACAGCCGCTTCGTCCGTGGTT	Amplify 3'*MoNAP1* downstream fragment
**MoNap1_downR**	ACGACGGCCAGTGCCAAGCTTGCCACGGCGTATTCTTGAATCCTG	Amplify 3'*MoNAP1* downstream fragment
**MoNap1_pYF11_F**	ACTCACTATAGGGCGAATTGGGTACTCAAATTGGTTATACCTCCATGTTCATCTCGGGCA	Amplify *MoNAP1* Complemented fragment
**MoNap1_pYF11_R**	CACCACCCCGGTGAACAGCTCCTCGCCCTTGCTCACGCTCTGCTTGCACTCGGCGGC	Amplify *MoNAP1* Complemented fragment
**MoNap1_P1**	CCATTCTGCCATACCTGACTGACGAATC	Verify 5'*MoNAP1* upstream fragment
**MoNap1_P2**	CACCGCCTGGACGACTAAACCA	Verify 5'*MoNAP1* upstream fragment
**MoNap1_P3**	AAAAACTTACGGCGCGATAGGGTCG	Verify 5'*MoNAP1* upstream fragment
**MoNap1_P4**	ACAGGACGATGCTTCCAAGCCGA	Verify 3'*MoNAP1* downstream fragment
**MoNap1_P5**	CCAGCACTCGTCCGAGGGCAAA	Verify 3'*MoNAP1* downstream fragment
**MoNap1_P6**	GTGGCGAGAAAGTTGCGGAAGGT	Verify 3'*MoNAP1* downstream fragment
**MoNap1_RT_F**	CCCAACAGCCTGGTGTTGACAGTATC	RT-PCR of *MoNAP1*
**MoNap1_RT_R**	TGCTCATCGCGATCGGTGATCA	RT-PCR of *MoNAP1*
**Tubulin_RT_F**	CCAGCCTTCAGTCCTGGGTC	RT-PCR of *MoTUBULIN*
**Tubulin_RT_R**	AGGGCAGTGATCTCCTTCTG	RT-PCR of *MoTUBULIN*
**MoNap1_TZ_F**	CTACTCCATACTTTGGGCGGGTTGGTA	Verify probe fragment
**MoNap1_TZ_R**	CGGTCCTACTGCAGATGACCTCTTCTCT	Verify probe fragment
**MoAlb1_qrt_F**	TGACACCTTCCTCAACACC	qPCR of *MoALB1*
**MoAlb1_qrt_R**	CGAGCCAGATTTA AGCAGCC	qPCR of *MoALB1*
**MoBuf1_qrt_F**	TAC AAGCACCTCGAGATTGG	qPCR of *MoBUF1*
**MoBuf1_qrt_R**	CAGTA ATCTTCTTGTCGGCC	qPCR of *MoBUF1*
**MoRsy1_qrt_F**	TTCCTCGACA AGCTCTGG	qPCR of *MoRSY1*
**MoRsy1_qrt_R**	AGGGCAGTGATCTCCTTCTG	qPCR of *MoRSY1*
**MoZfp6_qrt_F**	GTGTGGCATCTGACCGCATGGCT	qPCR of *MoZFP6*
**MoZfp6_qrt_R**	ACGAACGCAAAGGTTTGCCTGTGGC	qPCR of *MoZFP6*
**MoDit2_qrt_F**	TTTGGTCCGACACCCAAAGAC	qPCR of *MoDIT2*
**MoDit2_qrt_R**	GTGGCCGTAGACGTCCTGCC	qPCR of *MoDIT2*
**Tubulin_qrt_F**	CCAGCCTTCAGTCCTGGGTC	qPCR of *MoTUBULIN*
**Tubulin_qrt_R**	AGGGCAGTGATCTCCTTCTG	qPCR of *MoTUBULIN*
**MoCdc28_BDF**	ATGGCCATGGAGGCCGAATTCATGGAAAACTACCAGAAGCTCGA	Amplify *MoCDC28* cDNA fragment
**MoCdc28_BDR**	CCGCTGCAGGTCGACGGATCCCTATCGCCTCGGGGGCGC	Amplify *MoCDC28* cDNA fragment
**MoGin4_BDF1**	ATGGCCATGGAGGCCGAATTCATGGAAGTTCGTCCCCAGGC	Amplify *MoGIN4* cDNA fragment
**MoGin4_BDR1**	TGAAACTTCCTGCTGCTCTGAACTGAGCTAGC	Amplify *MoGIN4* cDNA fragment
**MoGin4_BDF2**	CAGAGCAGCAGGAAGTTTCATGCTCGCTCG	Amplify *MoGIN4* cDNA fragment
**MoGin4_BDR2**	CCGCTGCAGGTCGACGGATCCTCACGAATTCAAGGTTTTGATCA	Amplify *MoGIN4* cDNA fragment
**MoClb1_BDF**	ATGGCCATGGAGGCCGAATTCATGGCTTATCACCACCACAGCA	Amplify *MoCLB1* cDNA fragment
**MoClb1_BDR**	CCGCTGCAGGTCGACGGATCCTCATAAGTACGAGCCGTGGTTTG	Amplify *MoCLB1* cDNA fragment
**MoNbp2_BDF**	ATGGCCATGGAGGCCGAATTCATGATGGCTGTCTTGTCTCAAAGTG	Amplify *MoNBP2* cDNA fragment
**MoNbp2_BDR**	CCGCTGCAGGTCGACGGATCCTCACCGGCTCTTGCGATCT	Amplify *MoNBP2* cDNA fragment
**MoAts1_BDF**	ATGGCCATGGAGGCCGAATTCATGGACTTCGCATTTGCCC	Amplify *MoATS1* cDNA fragment
**MoAts1_BDR**	CCGCTGCAGGTCGACGGATCCTCAACTAGAAGGCTTTCCAATCATAA	Amplify *MoATS1* cDNA fragment

**MoCyc1_BDF**	ATGGCCATGGAGGCCGAATTCATGCCCCCAGCCCGGACT	Amplify *MoCYC1* cDNA fragment
**MoCyc1_BDR**	CCGCTGCAGGTCGACGGATCCCTACAGCTGGTCGATAGCTACGTC	Amplify *MoCYC1* cDNA fragment
**MoClb3_BDF**	ATGGCCATGGAGGCCGAATTCATGGACGCAAGGCCACAACGTC	Amplify *MoCLB3* cDNA fragment
**MoClb3_BDR**	CCGCTGCAGGTCGACGGATCCTTATGCTTGCGTCGCGGTGG	Amplify *MoCLB3* cDNA fragment
**MoNap1_ADF**	GCCATGGAGGCCAGTGAATTCATGGCCGAGCCCATTCCCAA	Amplify *MoNAP1* cDNA fragment
**MoNap1_ADR**	CAGCTCGAGCTCGATGGATCCCTAGCTCTGCTTGCACTCGGCG	Amplify *MoNAP1* cDNA fragment
**MoNbp2_upF**	GGTACCCGGGGATCCTCTAGAGTCTATTCTTACAACCCGTCCTTCCG	Amplify 5'*MoNBP2* upstream fragment
**MoNbp2_upR**	TTCATTGTTGACCTCCACTAGGAAACACTCGCGACCCATCAAT	Amplify 5'*MoNBP2* upstream fragment
**MoNbp2_downF**	GCAAAGGAATAGAGTAGATGTGGCGCAATACATCTGGAGTCA	Amplify 3'*MoNBP2* upstream fragment
**MoNbp2_downR**	ACGACGGCCAGTGCCAAGCTTGCAAAACCGACGGTGACAGCTT	Amplify 3'*MoNBP2* upstream fragment
**MoClb3_upF**	GGTACCCGGGGATCCTCTAGATCTTGCCCGCGGTTTTACTGGA	Amplify 5'*MoCLB3* upstream fragment
**MoClb3_upR**	TTCATTGTTGACCTCCACTATCAAATAGGCCGAGAGTTCGGCG	Amplify 5'*MoCLB3* upstream fragment
**MoClb3_downF**	GCAAAGGAATAGAGTAGATCACGTACATAATTTCGAGCCCAAGG	Amplify 3'*MoCLB3* upstream fragment
**MoClb3_downR**	ACGACGGCCAGTGCCAAGCTTCAGTGGTTATTGACTTGCCTATGCG	Amplify 3'*MoCLB3* upstream fragment
**MoNAP1_YFPCF**	CAATCACAATGGCCGGATCCATGGCCGAGCCCATTCCC	Amplify *MoNAP1* BiFC fragment
**MoNAP1_YFPCR**	CTTGCAGGCCGGGCGCCCGGGCTCTGCTTGCACTCGGC	Amplify *MoNAP1* BiFC fragment
**MoNBP2_YFPNF**	GTCTATATCATGGCCTCTAGAATGATGGCTGTCTTGTCTCAAAGTG	Amplify *MoNBP2* BiFC fragment
**MoNBP2_YFPNR**	GTCGCTTACTGCAGGTCGACCCGGCTCTTGCGATCT	Amplify *MoNBP2* BiFC fragment
**MoCLB1_YFPNF**	GTCTATATCATGGCCTCTAGAATGGCTTATCACCACCACAGCA	Amplify *MoCLB1* BiFC fragment
**MoCLB1_YFPNR**	GTCGCTTACTGCAGGTCGACTAAGTACGAGCCGTGGTTTG	Amplify *MoCLB1* BiFC fragment
**MoCLB3_YFPNF**	GTCTATATCATGGCCTCTAGAATGGACGCAAGGGTAAGCGCA	Amplify *MoCLB3* BiFC fragment
**MoCLB3_YFPNR**	GTCGCTTACTGCAGGTCGACTGCTTGCGTCGCGGTGG	Amplify *MoCLB3* BiFC fragment
