# Supplementary material for: MoNap1, a Nucleosome Assemble Protein 1, Regulates Growth, Development, and Pathogenicity in Magnaporthe oryzae
Source: J Fungi (Basel). 2022 Dec 28;9(1):50. doi: 10.3390/jof9010050 (PMC9862126; doi:10.3390/jof9010050)
Supplement: Supplementary file 1 [file jof-09-00050-s001.zip › jof-2089421-SI/Table S2. Differentially expressed genes related to melanin biosynthesis and pathogenesis in ΔMonap1 mutant.pdf]

Table S2. Differentially expressed genes related to melanin biosynthesis and pathogenesis in *ΔMonap1* mutant using |log_2FoldChange| values>1.5 and Padj<0.05

Gene ID	Description	Log₂-fold Change	Name
	**Pathogenesis**		
MGG_04404	pisatin demethylase	-13.53	*PDA*
MGG_07404	tripeptidyl-peptidase 1	-7.46	*TPP1*
MGG_15508	hypothetical protein	-4.07	*ZFP6*
MGG_16196	hypothetical protein	-3.40	*QDR3*
MGG_07626	cytochrome P450 monooxygenase	-3.09	*DIT2*
MGG_06872	hypothetical protein	-2.96	*YEP1*
MGG_08519	aflatoxin B1 aldehyde reductase member 3	-2.66	*ALR1*
MGG_00863	hypothetical protein	-2.49	*SCW4*
MGG_06772	hypothetical protein	-2.31	*PRY2*
MGG_12773	aminopeptidase Y	-1.73	*APE3*
MGG_01336	bacteriodes thetaiotaomicron symbiotic chitinase	-1.72	*CTS2*
MGG_13177	hypothetical protein	-1.55	*SOD1*
MGG_09246	protease	1.52	*PRB1*
MGG_09322	hypothetical protein	1.66	*PLP*
MGG_08850	cAMP-independent regulatory protein pac2	1.90	*MIT1*
MGG_04699	C2H2 finger domain-containing protein FlbC	2.05	*FLBC*
MGG_12442	aminopeptidase Y	2.31	*APE3*
MGG_07982	cytochrome P450 monooxygenase	2.32	*DIT2*
MGG_00681	hypothetical protein	2.44	*COQ5*
MGG_00416	MFS transporter	5.52	*TPO1*
MGG_10671	pentalenene synthase	6.95	*PSS*
	**Melanin biosynthesis**		
MGG_07219	conidial yellow pigment biosynthesis polyketide synthase	-4.90	*ALB1*
MGG_13464	laccase	-4.75	*LAC*
MGG_07216	versicolorin reductase	-4.30	*STCU2*
MGG_02252	tetrahydroxynaphthalene reductase	-3.22	*BUF1*
MGG_05059	scytalone dehydratase	-2.90	*SDH1*
MGG_07215	transcription factor Cmr1	-2.72	*CMR1*
