# Supplementary figures and images for: MoNap1, a Nucleosome Assemble Protein 1, Regulates Growth, Development, and Pathogenicity in Magnaporthe oryzae
Source: J Fungi (Basel). 2022 Dec 28;9(1):50. doi: 10.3390/jof9010050 (PMC9862126; doi:10.3390/jof9010050)

**Figure S1**

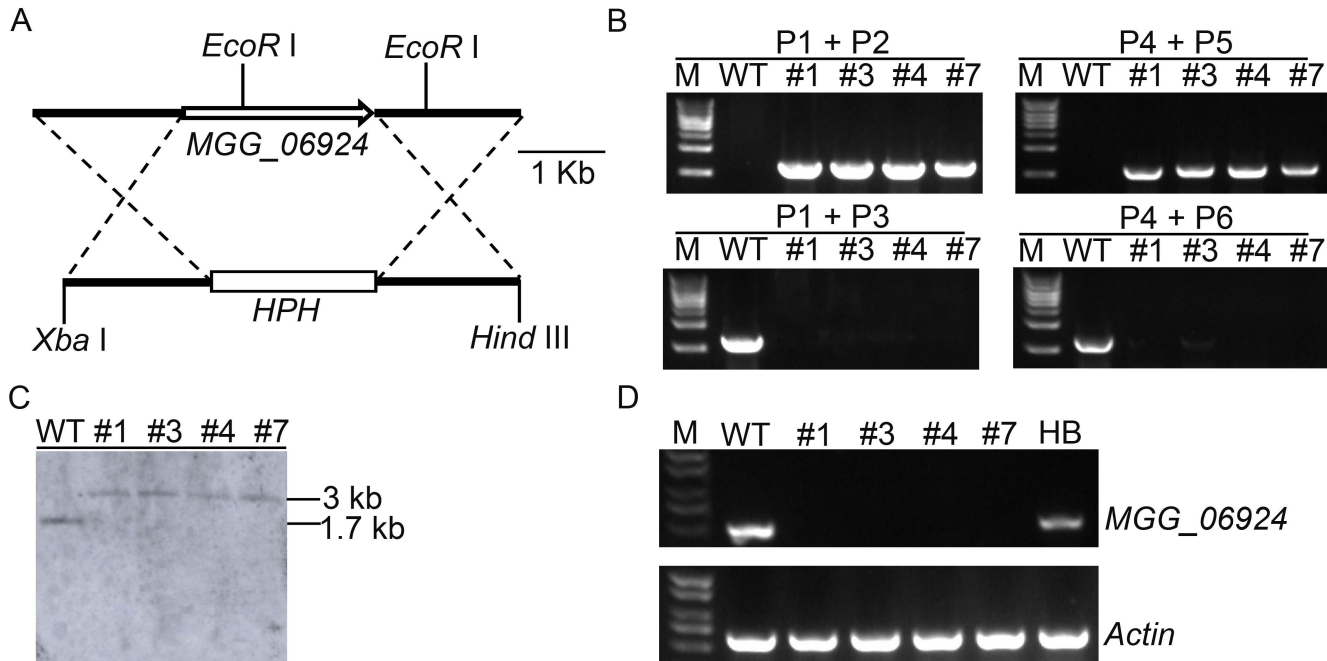

Supplement: Supplementary file 1 [file jof-09-00050-s001.zip › jof-2089421-SI/Fig.S1.pdf]

**Figure S2**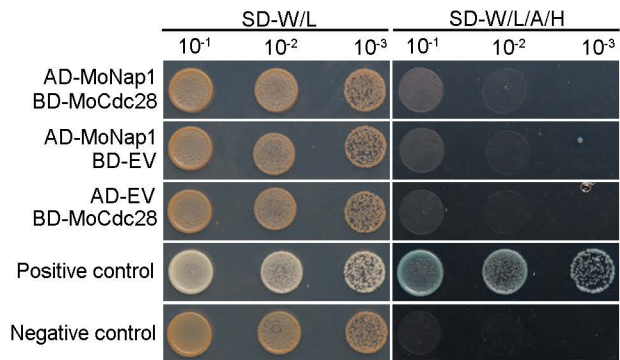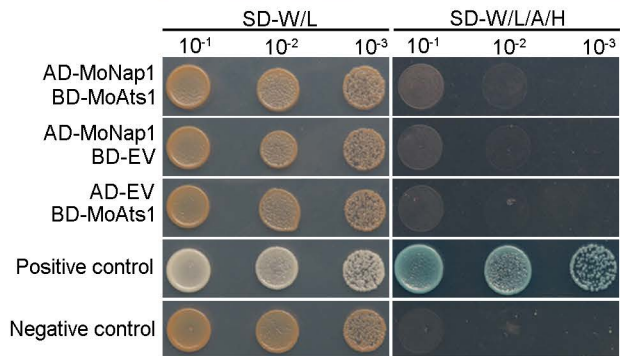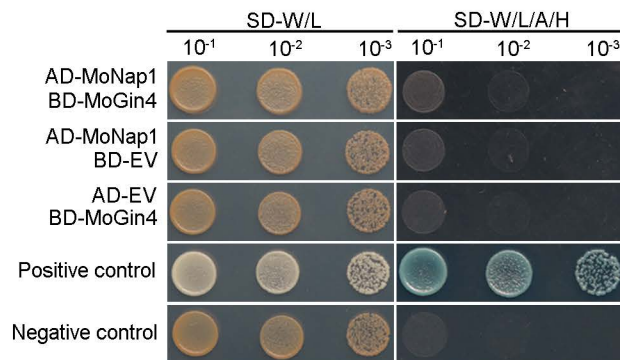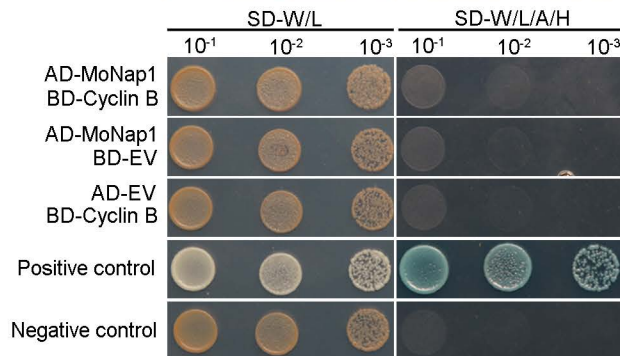

Supplement: Supplementary file 1 [file jof-09-00050-s001.zip › jof-2089421-SI/Fig.S2.pdf]

**Figure S4**

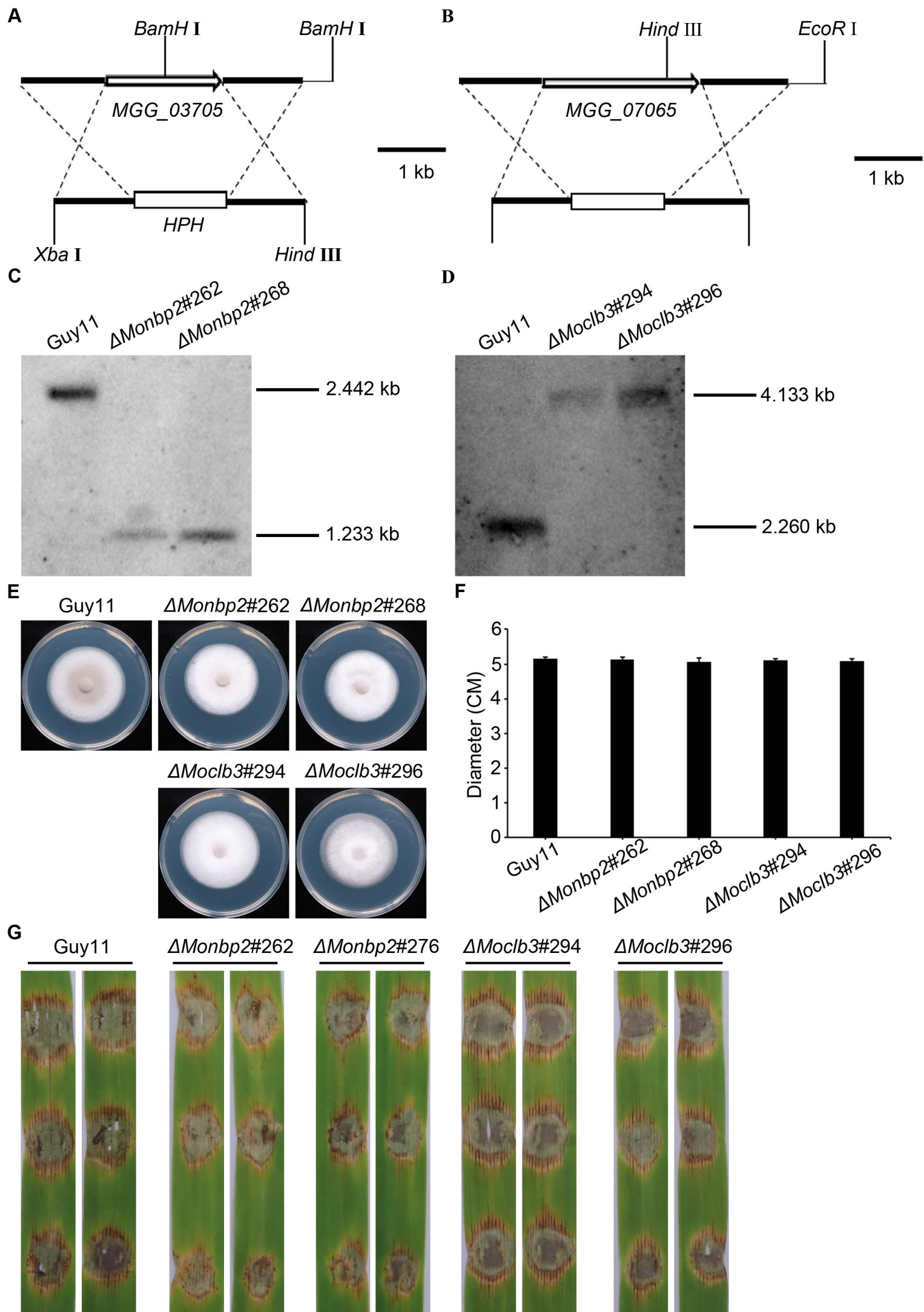

Supplement: Supplementary file 1 [file jof-09-00050-s001.zip › jof-2089421-SI/Fig.S4.pdf]

Figure S5

A

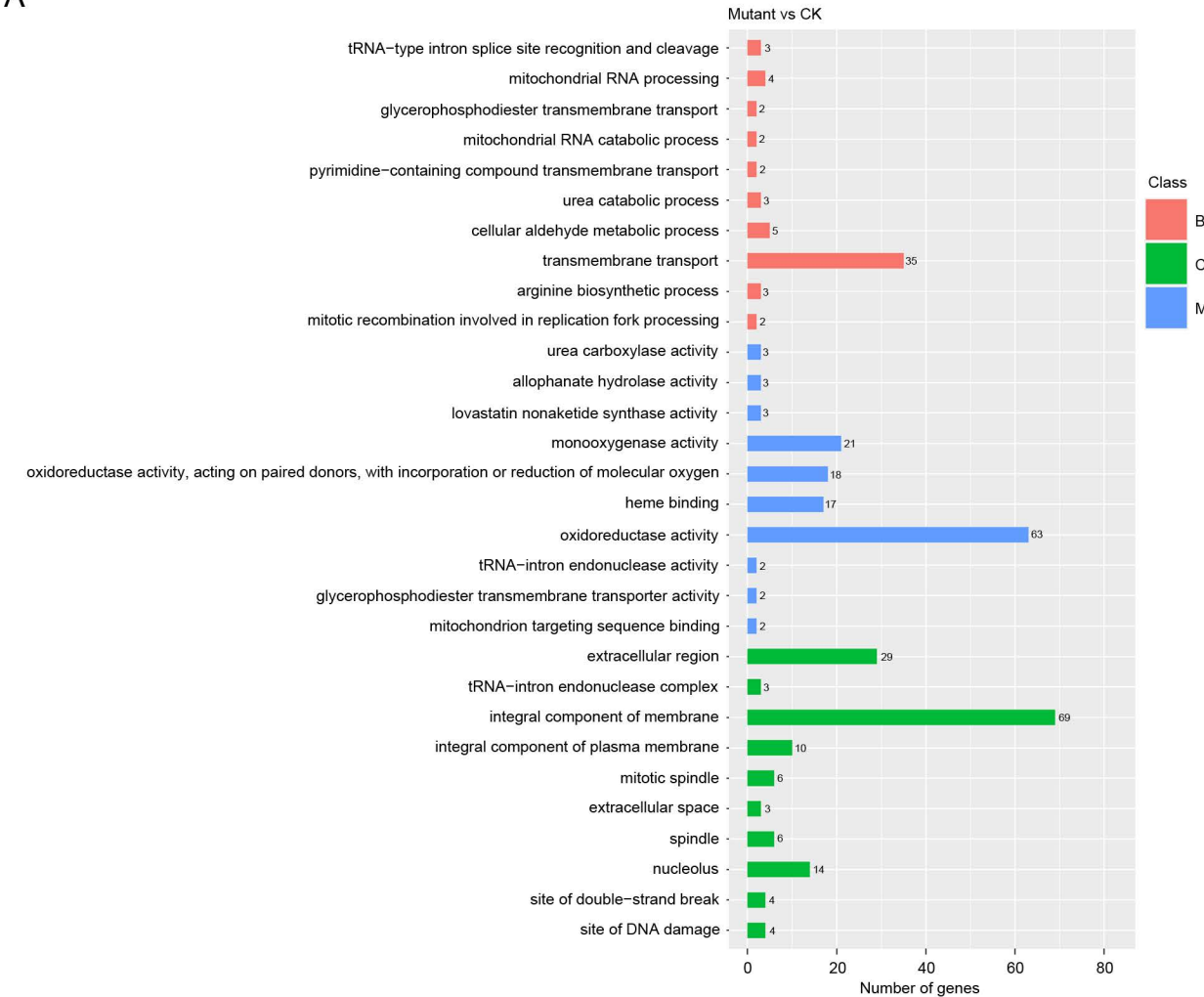

B

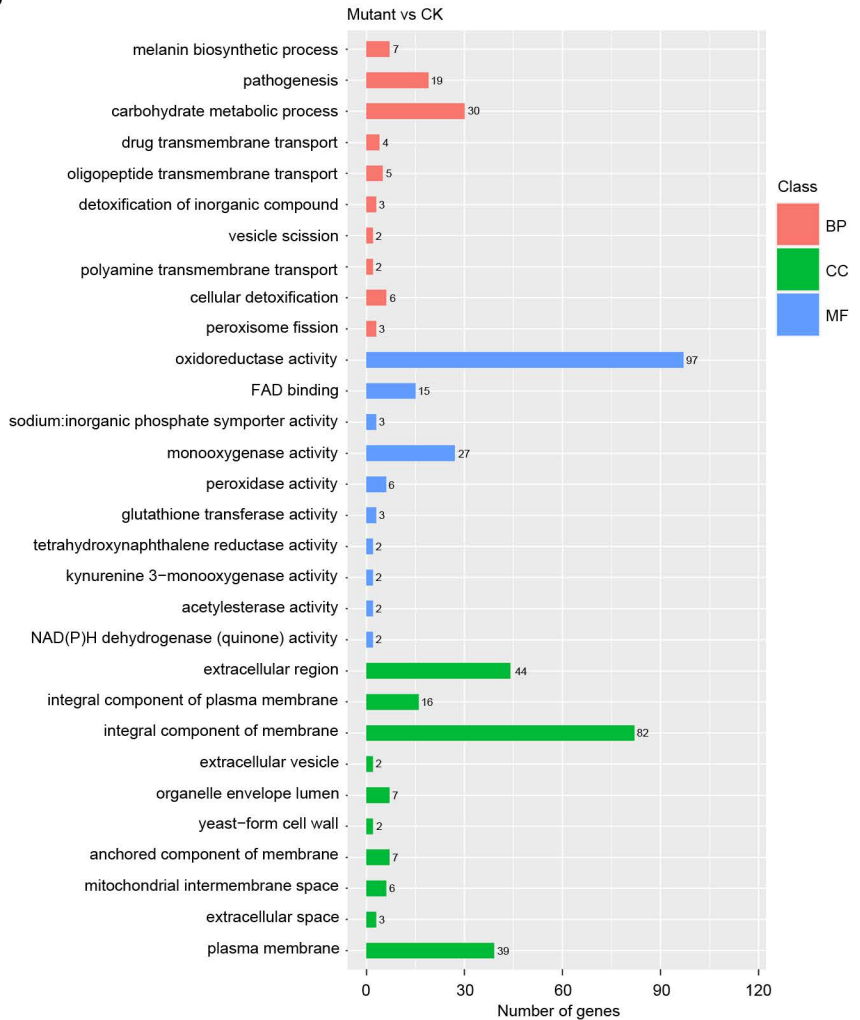

Supplement: Supplementary file 1 [file jof-09-00050-s001.zip › jof-2089421-SI/Fig.S5.pdf]

Figure S6

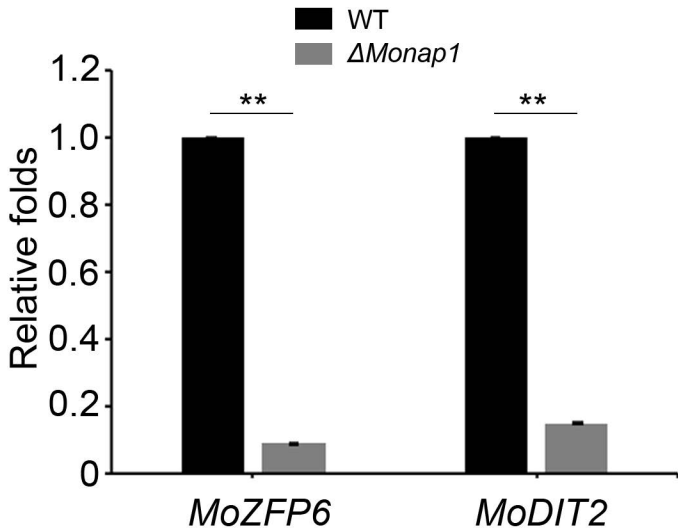

Supplement: Supplementary file 1 [file jof-09-00050-s001.zip › jof-2089421-SI/Fig.S6.pdf]

Figure S7

A

Pathway Name

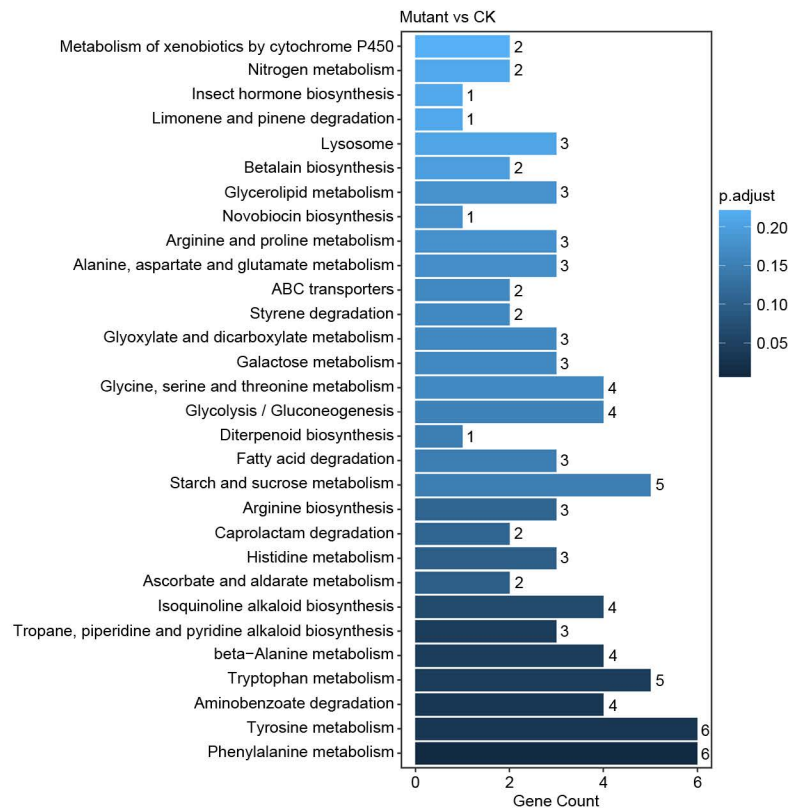

B

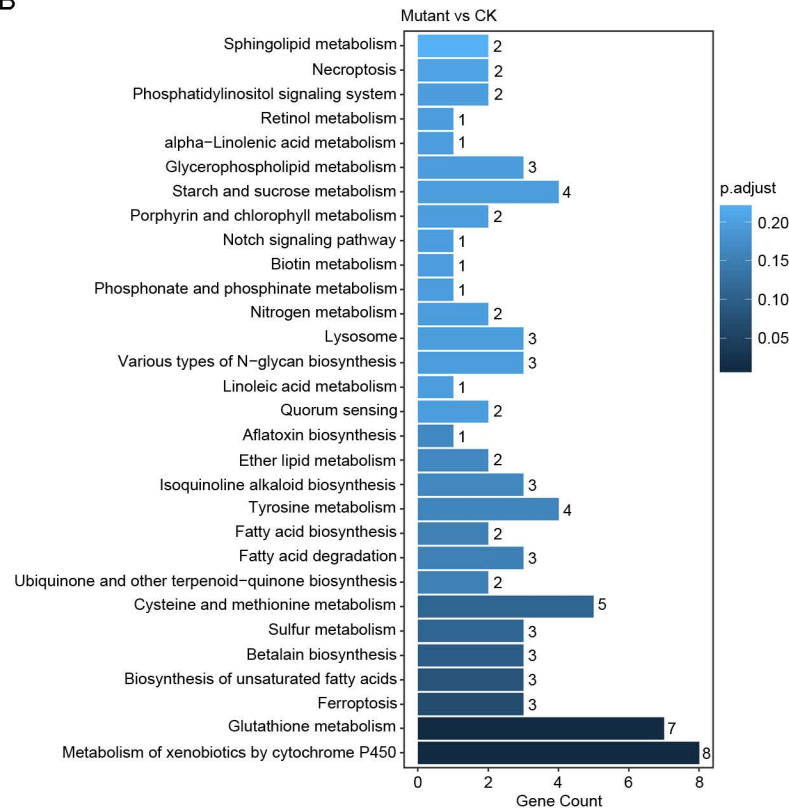

Supplement: Supplementary file 1 [file jof-09-00050-s001.zip › jof-2089421-SI/Fig.S7.pdf]
